# Exploring the Relationship Between Immune Cells and Scoliosis by Mendelian Randomization, Colocalization Analysis, and SMR

**DOI:** 10.1155/mi/8833556

**Published:** 2025-03-26

**Authors:** Chaofan Qin, Zhengjian Yan, Qingshuai Yu, Mingxin Chen, Tao Hu, Xin Wang, Bo Lei, Yu Chen, Ke Ma, Zhongliang Deng, Si Cheng

**Affiliations:** Department of Orthopedics, Second Affiliated Hospital, Chongqing Medical University, Chongqing, China

**Keywords:** cis-eQTL, colocalization analysis, immune cells, Mendelian randomization, scoliosis, SMR

## Abstract

**Background:** Scoliosis is a condition that can have severe consequences for millions of individuals on an annual basis. Current research in this field is increasingly focusing on the role of the immune system in the development of the disease. However, the precise relationship between immunity and scoliosis remains to be fully elucidated.

**Method:** Our investigation involved a comprehensive Mendelian randomization (MR) analysis to explore the potential causal relationship between immune cells and scoliosis. The comprehensive univariable MR analysis encompassed 731 immune cells to explore their relationship with scoliosis. Cochran's *Q* test, the leave-one-out test, and MR-Egger intercept analysis were used to assess pleiotropy and heterogeneity. We performed multivariable MR analysis to account for potential confounding factors between the immune cells. The colocalization analysis and summary data-based MR (SMR) analysis were utilized to explore relationship between immune cells and cis-eQTL.

**Results:** Our study identified 13 immune cells that were significantly associated with scoliosis by univariable MR, including four risk factors and nine protective factors for scoliosis. In order to reduce confounding between immune cells, multivariable MR was employed, and it was determined that only six immune cell types had independent effects on scoliosis. *SERPINH1* shared the same variant with CX3CR1 on CD14− CD16−. *FSD1L* shared the same variant with CCR2 on CD14− CD16−. *SNHG14*, *SNORA33*, *NET1*, and *SNORD100* shared the same variant with HLA DR on CD14+ CD16+ monocyte.

**Conclusion:** Our findings suggested a possible link between immune cells and scoliosis and found the key genes for the immune cell, which provides a new direction for further research. However, the specific underlying mechanisms require further investigation in future experiments.

## 1. Introduction

Scoliosis, a three-dimensional deformation of the spine, is characterized by a spinal curvature surpassing 10° in the Cobb angle [1]. As scoliosis progresses, it may lead to back pain, diminished cardiorespiratory function, depression, and changes in body image and can result in mortality in severe cases. These effects can significantly affect a patient's daily life [2]. Scoliosis is a multifactorial condition influenced by genetics, spine biomechanics, neurology, musculoskeletal factors, hormones, and other factors [3]. However, the precise etiology remains unknown.

In patients with scoliosis, a variety of alterations in the paravertebral muscles are observed, including myocyte necrosis and muscle fibrosis [4]. Additionally, previous studies have indicated the presence of increased infiltration and necrotic immune cells surrounding the paravertebral muscles [5]. A growing number of studies suggest that the immune system plays a significant role in scoliosis [6, 7]. Fibrosis results from the interaction between the immune system and the tissue [8, 9]. This finding underscores the vital role of the immune system in scoliosis. Nevertheless, the connection between immune cells and scoliosis is not yet fully understood because of the numerous confounding factors.

Mendelian randomization (MR) relies on the principles of the Mendelian independent distribution law and utilizes genotype information as an instrumental variable (IV) [10]. MR enables the exploration of causal relationships between exposure and outcome while effectively mitigating the impact of reverse causality and other confounding factors [10]. MR has gained popularity for investigating the causal relationships between exposures and outcomes. The concept of colocalization analysis has been proposed as a method for identifying genetic drivers that are common across multiple omics [11]. In cases where the overlap of association signals in two or more omics is attributable to the sharing of a causal variant, a phenomenon known as colocalization positivity, the probability of the genetic variant being a causal variant is significantly enhanced [11]. Summary data-based MR (SMR) can test for pleiotropic association between the expression level of a gene and the complex trait by using summary-level data from genome-wide association study (GWAS) and expression quantitative trait loci (eQTL) studies [12]. The SMR and heterogeneity in dependent instruments (HEIDI) methodology can be interpreted as an analysis to test if the effect size of a single-nucleotide polymorphism (SNPs) on the phenotype is mediated by gene expression [12].

In our study, we aimed to use MR to elucidate the involvement of immune cells in scoliosis. Meanwhile, we utilized the colocalization analysis and SMR to explore the relationship between the immune cells and cis-eQTL. We expect to illuminate the link between immune cells and scoliosis, providing new insights into the treatment and prevention of this condition.

## 2. Method and Materials

### 2.1. Study Design

**Figure 1:**
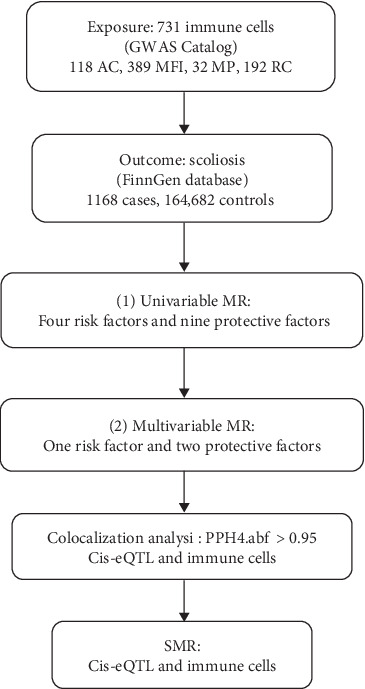
Schematic presentation of our study.

The analysis process was illustrated in Figure 1. The genetic variations chosen as IVs for MR meet three fundamental assumptions: (1) the selected IVs have a strong association with the immune cells; (2) the selected IVs have no correlation with potential confounding factors; and (3) the effect of IV on the outcome is exclusively through exposure [13, 14]. We performed univariable MR analysis to investigate the association between immune cells and scoliosis. In addition, univariable positive immune cells were used as covariates because there were interactions between immune cells. Multivariable MR was used to explore the independent effect values of univariable MR-positive immune cell cells on scoliosis [15]. We also performed colocalization analysis and SMR to find the key gene for the regulation of immune cells.

### 2.2. Data Source

GWAS data for immune cells were acquired from the latest study involving 3757 individuals of Sardinian descent within the European population [16]. The subjects included in this study were distributed as follows according to their sex: 43% of subjects were male and 57% were female, with ages ranging from 18 to 102 years [16]. A total of 731 immunophenotypes were included in the study, including 118 absolute cell (AC) counts, 389 median fluorescence intensities (MFIs) reflecting surface antigen levels, 32 morphological parameters (MPs), and 192 relative cell (RC) counts [16]. The study encompassed 22 million genetic variations [16].

Data on scoliosis for MR analysis were obtained from the FinnGen database (https://www.finngen.fi/fi). The data set comprised 1168 cases and 164,682 controls for scoliosis. Additionally, the data set contained 16,380,270 SNPs. The diagnosis of scoliosis is made in accordance with the International Classification of Diseases (ICD), encompassing both the ICD-10 (M41) and the ICD-9 (7373) classifications. All patients and controls included in the database are from Europe. Further detailed information can be found by visiting the FinnGen website (https://risteys.finngen.fi/endpoints/M13_SCOLIOSIS).

The eQTL generation (eQTLGen) dataset comprises 16,987 genes derived from blood samples of 31,684 European individuals. The eQTL data were obtained from the eQTLGen Consortium (https://eqtlgen.org/cis-eqtls.html). The comprehensive account of the data can be found in the original article [17].

### 2.3. MR

#### 2.3.1. Selection of IVs

The following criteria were employed for the screening of SNPs: initially, in order to ensure a strong association with immune cells, genome-wide significant SNPs (*p* < 5 × 10^−8^) were selected as IVs for MR analyses [18]. Additionally, the linkage disequilibrium (LD) between SNPs was eliminated, as substantial LD could introduce bias (*r*^2^ = 0.001, clumping distance = 10,000 kb) [18]. Finally, IVs with *F*-statistics less than 10 were excluded from the analyses. The formula *F* = beta^2^/standard error^2^ (SE) was utilized to calculate the *F*-statistic [14, 19] (Supporting Information 1: Table S1).

#### 2.3.2. Statistical Analysis

In our study, the primary analytical method was the inverse-variance weighted (IVW). A *p*-value of less than 0.05 for IVW was deemed statistically significant. Additionally, we employed MR-Egger to assess the size of the intercept term, as IVW does not incorporate this term [20]. Furthermore, to ensure the stability of the results, we utilized the weighted median, weighted mode, and simple mode approaches [14].

The Cochran's *Q* test was used to explore whether there was heterogeneity in our results so that we could choose the correct model to calculate the effect value of exposure on outcomes [14, 21]. Furthermore, the MR-Egger intercept test was used to confirm the absence of pleiotropy. We chose the *p*-value of the MR-Egger intercept greater than 0.05 as the norm [22]. Ultimately, a leave-one-out test was conducted to investigate whether the result was affected by a single SNP [14].

All the MR analyses were performed using the “TwoSampleMR” (version 0.5.7) packages within the R statistical software (version 4.3.1).

### 2.4. Colocalization Analysis

The R package coloc was used to perform the colocalization analysis between the immune cells and cis-eQTLs [11]. These immune cells was confirmed by multivariable MR. Analyses were conducted using SNPs harmonized by the TwoSampleMR package with default prior probabilities: p1 = 1E−4, p2 = 1E−4, p12 = 1E−5 [11]. The predefined probabilities p1, p2, and p12 indicate the likelihood of a substantial link between the SNP in the test area and gene expression, immune cell function, or both.

The posterior probabilities derived from the colocalization analysis correspond to one of five hypotheses [11]:

1. PPH0: SNPs are not associated with either trait.
2. PPH1: SNPs are associated with gene expression, but not with the immune cell.
3. PPH2: SNPs are associated with the immune cell, but not with gene expression.
4. PPH3: SNPs are associated with the immune cell and gene expression, but they are driven by different SNPs.
5. PPH4: SNPs are associated with the immune cell and gene expression, and they are driven by the common SNPs.

The threshold for statistical significance in colocalization was set at PPH4 > 0.95.

### 2.5. SMR Analysis

We performed SMR analysis to generate effect estimates between the immune cells and cis-eQTLs [12]. Using summary level data from GWAS and cis-eQTL studies, we assessed the relationship between identified immune cells and gene expression levels. In addition, we used the SMR software package (V.1.03) to perform allele harmonization and analysis. The *p*-value threshold for SMR analysis was 0.05.

The HEIDI method is used to detect heterogeneity between dependent IVs, which can help differentiate between pleiotropy and linkage scenario [12]. The HEIDI test indicates that the association is caused by shared genetic variation if the *p*-value is greater than 0.05. The HEIDI test was also conducted by using the SMR software (V.1.03).

## 3. Result

### 3.1. MR

#### 3.1.1. Univariable MR

**Figure 2:**
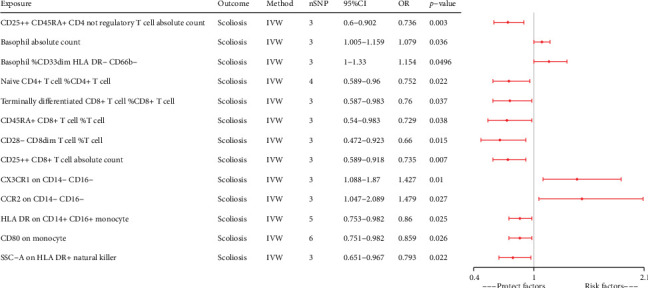
Forest plots of the effects of immune cells on scoliosis by using univariable MR.

We performed a comprehensive univariable MR analysis of 731 immune cells (Supporting Information 1: Table S2). We identified 40 immune cells with potential relationships to scoliosis. However, we excluded 27 immune cells because of insufficient SNP numbers for sensitivity analysis (Supporting Information 1: Table S3). Ultimately, we confirmed that 13 immune cells were linked to scoliosis, comprising four risk factors and nine protective factors (Figure 2).

#### 3.1.2. Multivariable MR

**Figure 3:**
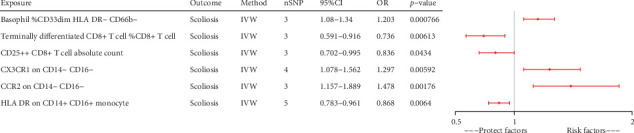
Forest plots of the effects of immune cells on scoliosis by using multivariable MR.

Because of the interactions between immune cells, we used multivariable MR to explore the independent effects of individual immune cells on scoliosis. All 13 immune cells used in the multivariable MR were identified by the univariable MR. Ultimately, our findings revealed that six immune cells remained associated with scoliosis, encompassing three risk factors and three protective factors (Figure 3). Basophil %CD33dim HLA DR− CD66b− (*p*-value, <0.001; OR, 1.203; 95%CI, 1.08−1.34), CX3CR1 on CD14− CD16− (*p*-value, 0.006; OR, 1.297; 95%CI, 1.078−1.562), and CCR2 on CD14− CD16− (*p*-value, 0.002; OR, 1.478; 95%CI, 1.157−1.889) could increase the risk of scoliosis. Terminally differentiated CD8+ T cell %CD8+ T cell (*p*-value, 0.006; OR, 0.736; 95%CI, 0.591−0.916), CD25++ CD8+ T cell absolute count (*p*-value, 0.043; OR, 0.836; 95%CI, 0.702−0.995), and HLA DR on CD14+ CD16+ (*p*-value, 0.006; OR, 0.868; 95%CI, 0.783−0.961) decrease the risk of scoliosis.

#### 3.1.3. Sensitivity Analysis

**Figure 4:**
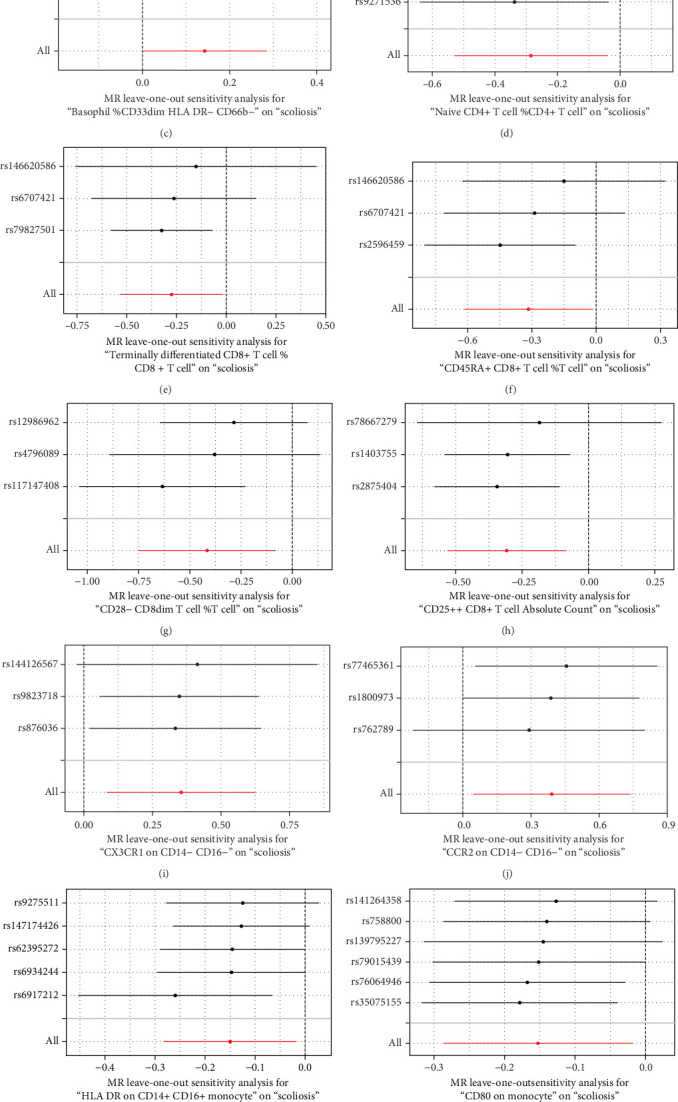
The leave-one-out plot of univariable MR. (A) CD25++ CD45RA+ CD4 not regulatory T cell absolute count; (B) Basophil absolute count; (C) Basophil %CD33dim HLA DR− CD66b−; (D) naive CD4+ T cell %CD4+ T cell; (E) terminally differentiated CD8+ T cell %CD8+ T cell; (F) CD45RA+ CD8+ T cell %T cell; (G) CD28− CD8dim T cell %T cell; (H) CD25++ CD8+ T cell absolute count; (I) CX3CR1 on CD14− CD16−; (J) CCR2 on CD14− CD16−; (K) HLA DR on CD14+ CD16+ monocyte; (L) CD80 on monocyte; (M) SSC-A on HLA DR+ natural killer.

Sensitivity analysis included the Cochran's *Q* test, MR-Egger intercept test, and the leave-one-out test. Cochran's *Q* test was used to examine the presence of heterogeneity. The *p*-values from Cochran's *Q* test were all greater than 0.05, indicating the absence of heterogeneity in our results (Table 1). Furthermore, no evidence of pleiotropy was observed in immune cells by using MR-Egger intercept test (Table 1). Finally, the leave-one-out test results suggested that our MR results were not influenced by any single SNP (Figure 4).

### 3.2. Colocalization Analysis

Colocalization analysis strongly suggested that *RP11-744N12.3* (PPH4.abf = 0.995), *IL2RA* (PPH4.abf = 0.994), *SAPCD1* (PPH4.abf = 0.994), *KIF11* (PPH4.abf = 0.986), *FBLN1* (PPH4.abf = 0.966), *PPRC1* (PPH4.abf = 0.965), *C20orf118* (PPH4.abf = 0.962), *CTD-3014M21.4* (PPH4.abf = 0.961), and *TFP1* (PPH4.abf = 0.952) shared the same variant with CD25++ CD8+ T cell absolute count (Table 2). *PAAF1* (PPH4.abf = 0.977) and *SERPINH1* (PPH4.abf = 0.966) shared the same variant with CX3CR1 on CD14− CD16− (Table 2). *FSD1L* (PPH4.abf = 0.972) shared the same variant with CCR2 on CD14− CD16− (Table 2). *SNHG14* (PPH4.abf = 0.987), *SNORA33* (PPH4.abf = 0.962), *NET1* (PPH4.abf = 0.961), and *SNORD100* (PPH4.abf = 0.958) shared the same variant with HLA DR on CD14+ CD16+ monocyte (Table 2).

### 3.3. SMR Analysis

The SMR was utilized to validate the result of the colocalization analysis (Table 2). *IL2RA* (P_SMR, <0.001; P_HIEDI, 0.001) was associated with CD25++ CD8+ T cell absolute count. *PAAF1* (P_SMR, <0.001; P_HIEDI, 0.001) and *SERPINH1* (P_SMR, <0.001; P_HIEDI, 0.295) were associated with CX3CR1 on CD14− CD16−. *FSD1L* (P_SMR, <0.001; P_HIEDI, 0.298) was associated with CCR2 on CD14− CD16−. *SNHG14* (P_SMR, <0.001; P_HIEDI, 0.349), *SNORA33* (P_SMR, <0.001; P_HIEDI, 0.249), *NET1* (P_SMR, <0.001; P_HIEDI, 0.816), and *SNORD100* (P_SMR, <0.001; P_HIEDI, 0.308) was associated with HLA DR on CD14+ CD16+ monocyte. However, The HIEDI test indicated that the association of *IL2RA* and *PAAF1* was caused by LD (P_HIEDI, <0.05).

## 4. Discussion

In this study, we confirmed that 13 immune cells were associated with scoliosis, encompassing four risk factors and nine protective factors, through univariable MR. Concurrently, we applied multivariable MR to control for confounding effects among the immune cells. Ultimately, our analysis substantiated the connection between six immune cells and scoliosis, comprising three protective factors and three risk factors. *SERPINH1* shared the same variant with CX3CR1 on CD14− CD16− which could increase the risk of scoliosis. *FSD1L* shared the same variant with CCR2 on CD14− CD16− which could increase the risk of scoliosis. *SNHG14*, *SNORA33*, *NET1*, and *SNORD100* shared the same variant with HLA DR on CD14+ CD16+ monocyte which could decrease the risk of scoliosis.

In our MR analysis, we noted that the increased expression of CX3CR1 on CD14− CD16−, which shared the same variant with *SERPINH1*, and CCR2 on CD14− CD16−, which shared the same variant with *FSD1L*, was associated with an elevated risk of scoliosis. It has been demonstrated that CX3CR1 deficiency facilitated muscle repair and rescued Ccl2−/− mice from impaired muscle regeneration [23]. Increased expression of apolipoprotein E (ApoE), which could stimulate macrophage phagocytosis, in CX3CR1-deficient mice promotes clearance of necrotic muscle fiber [23]. *SERPINH1* promotes the production of anti-inflammatory factors by M2 cells, thereby controlling the inflammatory balance [24]. We hypothesized that CX3CR1 may inhibit the function of *SERPINH1*, which could result in immune disorders and, ultimately, scoliosis. Controlling the balance between CX3CR1 and *SERPINH1* may be beneficial for intervention in scoliosis. CCR2 is a key factor that regulates immune cell recruitment and function and plays an important role in muscle regeneration and muscle damage repair [25]. Furthermore, myocyte repair is significantly reduced in CCR2 knockout mice [26]. It seems that CCR2 is a protective factor for muscle. This is contrary to our results. Further research on CCR2 is required. Meantime, the link between *FSD1L* and CCR2 still needs further study. This may provide new insights into early intervention for scoliosis.

In our study, we observed that HLA DR on CD14+ CD16+ monocyte was associated with a reduced risk of scoliosis, and *SNHG14*, *SNORA33*, and *NET1* shared the same variant with HLA DR on CD14+ CD16+ monocyte. CD14+ CD16+ monocyte is the intermediate monocyte (CD14+CD16+) [16]. Locally damaged muscle attracts monocytes, which then remove local inflammation and necrosis and then promote muscle differentiation and repair [27, 28]. *SNHG14* is able to inhibit the body's immune response to tumor, thereby promoting tumor immune tolerance and immune escape [29]. Studies have shown that *Netrin-1* is effective in inhibiting monocyte adhesion and also inhibits the production of inflammatory factors [30]. The *SNORA33* and *SNORD100* genes are members of the small nuclear ribonucleic acid (snoRNA) gene family [31]. SnoRNAs could affect the gene expression profile of immune cells and has the potential to impact the development, activation, and tolerance of these cells [31]. *SNHG14*, *SNORA33*, *NET1*, and *SNORD100* play different roles in immunity. Exploring their roles in scoliosis will help us to explore new therapeutic targets for scoliosis.

Our research indicated that the terminally differentiated CD8+ T cell %CD8+ T cell and CD25++ CD8+ T cell absolute count were associated with a reduced risk of scoliosis. CD8+ T cells comprise a variety of subtypes, including Tc1, Tc2, Tc9, Tc17, and CD8+ T regulatory (Treg) cells [32]. Tc9 cells and CD8+ Treg cells function primarily in immunosuppression [32]. Furthermore, CD25++ CD8+ T cells have been shown to possess immunosuppressive properties [33]. However, the protective role of CD8 in scoliosis still needs to be further explored.

Our results suggested that basophil %CD33dim HLA DR− CD66b− could increase the risk of scoliosis. Basophils can engage in the immune response within skeletal muscle through IL-6, a primary cytokine released by skeletal muscle [34]. The basophil count was markedly reduced in IL-6-deficient mice [34]. Therefore, we hypothesized that basophils might exert a risk role through IL-6 in the context of scoliosis.

Our MR analysis has several advantages. Firstly, we adopted a comprehensive approach, encompassing univariable and multivariable MR, to address potential confounding factors. Secondly, multiple sensitivity analyses were conducted to validate our hypotheses and mitigate bias. Thirdly, we restricted our study to the European population to minimize population bias. Finally, we utilized the SMR and colocalization analysis to explore the key gene of immune cells.

However, this study had several limitations. The generalizability of our conclusions to other populations is limited, as all of our data originate from European sources. The relatively small sample size introduces the potential for bias, necessitating larger sample sizes for more robust results. Despite employing univariable and multivariable MR, there remains the possibility of unmeasured pleiotropy. Additionally, we did not choose to apply Bonferroni correction in our analysis, as our primary goal was to identify potential therapeutic and prophylactic targets associated with scoliosis. Bonferroni's criteria are too strict and may have led to the exclusion of meaningful indicators in our context. Although this would increase the probability of type I errors, we used univariable and multivariable MR to reduce the occurrence of false positives.

## 5. Conclusion

Our MR analysis revealed 13 immune cells associated with scoliosis risk through univariable MR. Following adjustment for confounding effects between immune cells in our multivariable MR, we identified six immune cells as independent factors for scoliosis, including three risk factors and three protective factors. Meantime, we found that six genes associated with three immune cells. These six immune cells could potentially serve as biomarkers for scoliosis and provide novel insights into its treatment and prevention. However, further experiments are necessary to elucidate the underlying mechanisms.

## Figures and Tables

**Table 1:** The sensitivity analyses of the univariable MR.

Exposure	Egger_intercept	*p*-Value	*Q*	*Q*_*p*-value
CD25++ CD45RA+ CD4 not regulatory T cell absolute count	0.009	0.887	0.050	0.975
Basophil absolute count	0.065	0.427	1.594	0.451
Basophil %CD33dim HLA DR− CD66b−	−0.014	0.903	0.137	0.934
Naive CD4+ T cell %CD4+ T cell	0.044	0.534	1.256	0.740
Terminally differentiated CD8+ T cell %CD8+ T cell	0.037	0.800	2.199	0.333
CD45RA+ CD8+ T cell %T cell	0.051	0.509	1.929	0.381
CD28− CD8dim T cell %T cell	−0.131	0.359	2.540	0.281
CD25++ CD8+ T cell absolute count	0.053	0.568	0.777	0.678
CX3CR1 on CD14− CD16−	0.026	0.795	0.113	0.945
CCR2 on CD14− CD16−	0.084	0.638	0.419	0.811
HLA DR on CD14+ CD16+ monocyte	−0.173	0.239	3.438	0.487
CD80 on monocyte	0.078	0.218	3.504	0.623
SSC-A on HLA DR+ natural killer	0.022	0.787	0.359	0.836

**Table 2:** The result of the colocalization analysis and SMR between cis-eQTL and immune cells.

cis-eQTL	Immune cells	Colocalization analysis	SMR	
		PPH4.abf	P_SMR	P_HEIDI
RP11-744N12.3	CD25++ CD8+ T cell absolute count	0.995	NA	NA
IL2RA		0.994	2.53E−06	1.31E−03
SAPCD1		0.994	NA	NA
KIF11		0.986	6.84E−01	6.10E−01
FBLN1		0.966	NA	NA
PPRC1		0.965	NA	NA
C20orf118		0.962	7.78E−01	1.50E−01
CTD-3014M21.4		0.961	9.32E−01	3.49E−01
TFP1		0.952	NA	NA

PAAF1	CX3CR1 on CD14− CD16−	0.977	4.12E−05	1.03E−03
SERPINH1		0.966	3.90E−05	2.95E−01

FSD1L	CCR2 on CD14− CD16−	0.972	4.67E−05	2.98E−01

SNHG14	HLA DR on CD14+ CD16+ monocyte	0.987	4.84E−05	3.49E−01
SNORA33		0.962	5.10E−05	2.49E−01
NET1		0.961	1.22E−04	8.16E−01
SNORD100		0.958	5.84E−05	3.08E−01

Abbreviation: NA, not available.

## Data Availability

The data that support the findings of this study are openly available in GWAS Catalog (https://www.ebi.ac.uk/gwas), IEU Open GWAS (https://gwas.mrcieu.ac.uk), eQTLGen Consortium (https://www.eqtlgen.org/cis-eqtls.html) and FinnGen database (https://www.finngen.fi/en).

## Supporting Information

Supporting Information 1 Table S1: Information on the univariable MR of SNPs and the *F*-statistic of SNPs.

Supporting Information 2 Table S2: The results of univariable MR between the 731 immune cells and scoliosis.

Supporting Information 3 Table S3: The significant results of univariable MR between the 40 immune cells and scoliosis.
