# Supplementary material for: Exploring the Relationship Between Immune Cells and Scoliosis by Mendelian Randomization, Colocalization Analysis, and SMR
Source: Mediators Inflamm. 2025 Mar 26;2025:8833556. doi: 10.1155/mi/8833556 (PMC11964722; doi:10.1155/mi/8833556)
Supplement: Supporting Information 1 — Table S1: Information on the univariable MR of SNPs and the F-statistic of SNPs. [file 8833556.f1.docx]

| SNP | beta.exposure | eaf.exposure | pos.exposure | chr.exposure | pval.exposure | se.exposure | exposure | F |
| --- | --- | --- | --- | --- | --- | --- | --- | --- |
| rs11124653 | -0.1861 | 0.5907 | 39112166 | 2 | 3.74197E-12 | 0.0267 | CD25++ CD45RA+ CD4 not regulatory T cell Absolute Count | 48.5814221 |
| rs116517602 | 1.722 | 0.0041 | 198922068 | 1 | 3.67282E-19 | 0.1913 | CD25++ CD45RA+ CD4 not regulatory T cell Absolute Count | 81.02823037 |
| rs12712610 | 0.2347 | 0.2972 | 38897249 | 2 | 2.14981E-16 | 0.02843 | CD25++ CD45RA+ CD4 not regulatory T cell Absolute Count | 68.15103563 |
| rs113243185 | -0.2104 | 0.1996 | 32585071 | 6 | 8.06102E-09 | 0.03639 | Naive CD4+ T cell %CD4+ T cell | 33.42930594 |
| rs12712610 | 0.1457 | 0.2969 | 38897249 | 2 | 1.17501E-08 | 0.02548 | Naive CD4+ T cell %CD4+ T cell | 32.69792973 |
| rs146620586 | 1.495 | 0.0032 | 198524264 | 1 | 1.012E-08 | 0.2604 | Naive CD4+ T cell %CD4+ T cell | 32.96100351 |
| rs9271536 | 0.2324 | 0.7798 | 32589978 | 6 | 3.59832E-12 | 0.03331 | Naive CD4+ T cell %CD4+ T cell | 48.67690782 |
| rs114590598 | 2.421 | 0.0016 | 185852065 | 1 | 9.84306E-09 | 0.4204 | Basophil Absolute Count | 33.16379059 |
| rs3865444 | -0.4855 | 0.2132 | 51727962 | 19 | 7.50931E-35 | 0.03861 | Basophil Absolute Count | 158.117109 |
| rs78744187 | -0.5045 | 0.0673 | 33754548 | 19 | 9.45584E-14 | 0.06722 | Basophil Absolute Count | 56.32812887 |
| rs115805162 | 0.2464 | 0.2099 | 161410253 | 1 | 2.85299E-13 | 0.03359 | SSC-A on HLA DR+ Natural Killer | 53.80980266 |
| rs569911 | -0.1723 | 0.4305 | 160798209 | 1 | 4.62402E-10 | 0.02756 | SSC-A on HLA DR+ Natural Killer | 39.08518109 |
| rs71632979 | -0.5652 | 0.2402 | 161526397 | 1 | 1.97879E-74 | 0.0301 | SSC-A on HLA DR+ Natural Killer | 352.5910752 |
| rs115805162 | -0.2265 | 0.2083 | 161410253 | 1 | 2.75296E-12 | 0.03229 | Myeloid Dendritic Cell Absolute Count | 49.20399012 |
| rs516847 | 0.1559 | 0.4297 | 160799360 | 1 | 3.02197E-09 | 0.02621 | Myeloid Dendritic Cell Absolute Count | 35.38002932 |
| rs71632979 | 0.6546 | 0.2449 | 161526397 | 1 | 2.2909E-112 | 0.02797 | Myeloid Dendritic Cell Absolute Count | 547.7306819 |
| rs75072970 | 0.3859 | 0.0531 | 161058351 | 1 | 1.93999E-11 | 0.05732 | Myeloid Dendritic Cell Absolute Count | 45.32493539 |
| rs115805162 | -0.2673 | 0.2083 | 161410253 | 1 | 1.29211E-15 | 0.03328 | CD86+ myeloid Dendritic Cell Absolute Count | 64.51062987 |
| rs191217950 | 0.7582 | 0.0096 | 160940671 | 1 | 8.13093E-09 | 0.1312 | CD86+ myeloid Dendritic Cell Absolute Count | 33.39641815 |
| rs71632979 | 0.8088 | 0.2449 | 161526397 | 1 | 9.7051E-166 | 0.02785 | CD86+ myeloid Dendritic Cell Absolute Count | 843.3966782 |
| rs71639910 | 0.3667 | 0.0686 | 161937774 | 1 | 2.25476E-12 | 0.05205 | CD86+ myeloid Dendritic Cell Absolute Count | 49.63412296 |
| rs75072970 | 0.4059 | 0.0531 | 161058351 | 1 | 5.96898E-12 | 0.05879 | CD86+ myeloid Dendritic Cell Absolute Count | 47.66846665 |
| rs117147408 | 0.4819 | 0.0273 | 101980039 | 6 | 1.46599E-09 | 0.07946 | CD28- CD8dim T cell %T cell | 36.78042398 |
| rs12986962 | -0.1604 | 0.2827 | 111808558 | 2 | 1.54099E-08 | 0.02829 | CD28- CD8dim T cell %T cell | 32.14717638 |
| rs4796089 | -0.2225 | 0.1343 | 33814758 | 17 | 3.91201E-09 | 0.0377 | CD28- CD8dim T cell %T cell | 34.83191326 |
| rs139795227 | -1.147 | 0.0481 | 92842367 | 1 | 2.33776E-72 | 0.06195 | CD80 on monocyte | 342.8026846 |
| rs141264358 | -0.5868 | 0.016 | 90530314 | 1 | 4.18196E-08 | 0.1067 | CD80 on monocyte | 30.24484558 |
| rs35075155 | -0.1646 | 0.4402 | 53273195 | 3 | 7.76497E-09 | 0.02843 | CD80 on monocyte | 33.52014915 |
| rs758800 | 0.6619 | 0.8956 | 52529266 | 3 | 5.12153E-48 | 0.04462 | CD80 on monocyte | 220.0521572 |
| rs76064946 | -0.35 | 0.1204 | 93926697 | 1 | 2.60976E-16 | 0.04248 | CD80 on monocyte | 67.88394494 |
| rs79015439 | -0.7914 | 0.0126 | 94803124 | 1 | 6.94065E-11 | 0.1209 | CD80 on monocyte | 42.84888153 |
| rs1403755 | 0.2696 | 0.0864 | 30263492 | 18 | 8.818E-09 | 0.04674 | CD25++ CD8+ T cell Absolute Count | 33.27073227 |
| rs2875404 | -0.1537 | 0.657 | 76674553 | 3 | 3.159E-08 | 0.02772 | CD25++ CD8+ T cell Absolute Count | 30.74406454 |
| rs78667279 | -0.7492 | 0.0096 | 54957709 | 2 | 1.323E-08 | 0.1315 | CD25++ CD8+ T cell Absolute Count | 32.45966488 |
| rs144126567 | 0.5108 | 0.1735 | 161510516 | 1 | 5.50554E-57 | 0.03154 | CX3CR1 on CD14- CD16- | 262.2879865 |
| rs876036 | 0.1655 | 0.2589 | 50307710 | 7 | 5.06699E-09 | 0.02824 | CX3CR1 on CD14- CD16- | 34.34524442 |
| rs9823718 | -0.2149 | 0.1319 | 39293757 | 3 | 3.24198E-09 | 0.03622 | CX3CR1 on CD14- CD16- | 35.20269712 |
| rs146620586 | 1.455 | 0.0032 | 198524264 | 1 | 1.003E-08 | 0.2533 | CD45RA+ CD8+ T cell %T cell | 32.99556786 |
| rs2596459 | 0.1499 | 0.5099 | 31417540 | 6 | 1.652E-08 | 0.02649 | CD45RA+ CD8+ T cell %T cell | 32.02132873 |
| rs6707421 | 0.1592 | 0.3011 | 38897781 | 2 | 8.10588E-11 | 0.02442 | CD45RA+ CD8+ T cell %T cell | 42.50057518 |
| rs7583259 | 0.2378 | 0.5023 | 38921934 | 2 | 1.63606E-26 | 0.02213 | CD45RA+ CD8+ T cell %T cell | 115.4677951 |
| rs146620586 | 1.949 | 0.0032 | 198524264 | 1 | 5.29541E-12 | 0.2815 | Terminally Differentiated CD8+ T cell %CD8+ T cell | 47.93656162 |
| rs6707421 | 0.246 | 0.3011 | 38897781 | 2 | 1.20587E-19 | 0.02696 | Terminally Differentiated CD8+ T cell %CD8+ T cell | 83.25885585 |
| rs7583259 | 0.352 | 0.5023 | 38921934 | 2 | 2.38891E-46 | 0.02426 | Terminally Differentiated CD8+ T cell %CD8+ T cell | 210.5250281 |
| rs79827501 | -0.3142 | 0.0732 | 38431267 | 2 | 4.97279E-11 | 0.04765 | Terminally Differentiated CD8+ T cell %CD8+ T cell | 43.47970126 |
| rs147174426 | -0.3104 | 0.1692 | 161528012 | 1 | 2.64972E-20 | 0.03343 | HLA DR on CD14+ CD16+ monocyte | 86.21258595 |
| rs62395272 | -0.2623 | 0.2848 | 31394424 | 6 | 3.7653E-15 | 0.03321 | HLA DR on CD14+ CD16+ monocyte | 62.3819333 |
| rs6917212 | -0.5221 | 0.7769 | 32583005 | 6 | 4.09072E-58 | 0.03191 | HLA DR on CD14+ CD16+ monocyte | 267.7033328 |
| rs6934244 | 0.4143 | 0.1551 | 33024032 | 6 | 5.66239E-26 | 0.039 | HLA DR on CD14+ CD16+ monocyte | 112.8497633 |
| rs9275511 | 0.3664 | 0.6053 | 32674329 | 6 | 1.073E-35 | 0.02907 | HLA DR on CD14+ CD16+ monocyte | 158.8623075 |
| rs1800973 | -0.2492 | 0.0798 | 69744014 | 12 | 2.81598E-08 | 0.04479 | CCR2 on CD14- CD16- | 30.955224 |
| rs762789 | -0.1859 | 0.447 | 46402627 | 3 | 9.482E-14 | 0.02487 | CCR2 on CD14- CD16- | 55.87367136 |
| rs77465361 | 0.4573 | 0.0236 | 159009803 | 5 | 8.01807E-09 | 0.07909 | CCR2 on CD14- CD16- | 33.43176087 |
| rs3865444 | -0.5183 | 0.2132 | 51727962 | 19 | 1.14393E-40 | 0.03788 | Basophil %CD33dim HLA DR- CD66b- | 187.2157909 |
| rs4499342 | 0.2541 | 0.1527 | 8786913 | 19 | 3.85398E-08 | 0.04602 | Basophil %CD33dim HLA DR- CD66b- | 30.48709903 |
| rs7351079 | -0.2756 | 0.2722 | 51574038 | 19 | 1.36994E-13 | 0.03696 | Basophil %CD33dim HLA DR- CD66b- | 55.60252338 |
