# Supplementary material for: Exploring the Relationship Between Immune Cells and Scoliosis by Mendelian Randomization, Colocalization Analysis, and SMR
Source: Mediators Inflamm. 2025 Mar 26;2025:8833556. doi: 10.1155/mi/8833556 (PMC11964722; doi:10.1155/mi/8833556)
Supplement: Supporting Information 2 — Table S2: The results of univariable MR between the 731 immune cells and scoliosis. [file 8833556.f2.docx]

| exposure | method | nsnp | b | se | pval | or | or_lci95 | or_uci95 |
| --- | --- | --- | --- | --- | --- | --- | --- | --- |
| IgD+ B cell %B cell \|\| id:ebi-a-GCST90001391 | Wald ratio | 1 | -0.021 | 0.302 | 0.944 | 0.979 | 0.541 | 1.770 |
| IgD+ CD38+ B cell Absolute Count \|\| id:ebi-a-GCST90001392 | Inverse variance weighted | 2 | 0.073 | 0.670 | 0.913 | 1.076 | 0.289 | 4.003 |
| IgD+ CD38- B cell %B cell \|\| id:ebi-a-GCST90001395 | Inverse variance weighted | 2 | 0.296 | 0.288 | 0.305 | 1.344 | 0.764 | 2.362 |
| IgD+ CD38- B cell Absolute Count \|\| id:ebi-a-GCST90001396 | Wald ratio | 1 | 0.230 | 0.778 | 0.767 | 1.259 | 0.274 | 5.781 |
| Unswitched memory B cell %B cell \|\| id:ebi-a-GCST90001397 | Wald ratio | 1 | -0.241 | 0.303 | 0.425 | 0.786 | 0.434 | 1.422 |
| Unswitched memory B cell Absolute Count \|\| id:ebi-a-GCST90001398 | Inverse variance weighted | 2 | -0.280 | 0.310 | 0.366 | 0.756 | 0.412 | 1.386 |
| IgD- CD27- B cell %B cell \|\| id:ebi-a-GCST90001399 | Inverse variance weighted | 3 | -0.027 | 0.155 | 0.861 | 0.973 | 0.718 | 1.319 |
| IgD- CD27- B cell Absolute Count \|\| id:ebi-a-GCST90001401 | Inverse variance weighted | 2 | 0.025 | 0.146 | 0.866 | 1.025 | 0.770 | 1.364 |
| Plasma Blast-Plasma Cell %B cell \|\| id:ebi-a-GCST90001404 | Wald ratio | 1 | -0.272 | 0.450 | 0.546 | 0.762 | 0.315 | 1.842 |
| Memory B cell %B cell \|\| id:ebi-a-GCST90001406 | Wald ratio | 1 | 0.420 | 0.331 | 0.204 | 1.521 | 0.796 | 2.909 |
| Memory B cell Absolute Count \|\| id:ebi-a-GCST90001407 | Wald ratio | 1 | -0.294 | 0.676 | 0.664 | 0.745 | 0.198 | 2.805 |
| Naive-mature B cell %B cell \|\| id:ebi-a-GCST90001408 | Wald ratio | 1 | -0.441 | 0.347 | 0.204 | 0.643 | 0.326 | 1.271 |
| IgD- CD38+ B cell %B cell \|\| id:ebi-a-GCST90001410 | Wald ratio | 1 | 0.431 | 0.301 | 0.152 | 1.539 | 0.853 | 2.779 |
| IgD+ CD24+ B cell Absolute Count \|\| id:ebi-a-GCST90001412 | Wald ratio | 1 | -0.259 | 0.596 | 0.664 | 0.772 | 0.240 | 2.482 |
| IgD+ CD24- B cell %B cell \|\| id:ebi-a-GCST90001415 | Wald ratio | 1 | -0.149 | 0.167 | 0.375 | 0.862 | 0.621 | 1.197 |
| CD24+ CD27+ B cell %B cell \|\| id:ebi-a-GCST90001417 | Inverse variance weighted | 3 | 0.152 | 0.139 | 0.275 | 1.164 | 0.886 | 1.529 |
| CD24+ CD27+ B cell Absolute Count \|\| id:ebi-a-GCST90001418 | Wald ratio | 1 | -0.301 | 0.692 | 0.664 | 0.740 | 0.191 | 2.872 |
| CD20- CD38- B cell %B cell \|\| id:ebi-a-GCST90001422 | Wald ratio | 1 | 0.070 | 0.169 | 0.677 | 1.073 | 0.771 | 1.493 |
| IgD+ B cell %Lymphocyte \|\| id:ebi-a-GCST90001424 | Inverse variance weighted | 2 | -0.111 | 0.267 | 0.679 | 0.895 | 0.530 | 1.511 |
| IgD- CD38dim B cell %lymphocyte \|\| id:ebi-a-GCST90001426 | Inverse variance weighted | 2 | -0.578 | 0.231 | 0.012 | 0.561 | 0.357 | 0.882 |
| IgD+ CD38+ B cell %lymphocyte \|\| id:ebi-a-GCST90001429 | Wald ratio | 1 | 2.438 | 1.238 | 0.049 | 11.449 | 1.011 | 129.651 |
| IgD+ CD38- B cell %lymphocyte \|\| id:ebi-a-GCST90001431 | Inverse variance weighted | 2 | -0.053 | 0.492 | 0.914 | 0.948 | 0.361 | 2.488 |
| Unswitched memory B cell %lymphocyte \|\| id:ebi-a-GCST90001432 | Inverse variance weighted | 4 | -0.057 | 0.200 | 0.776 | 0.945 | 0.639 | 1.397 |
| IgD- CD27- B cell %lymphocyte \|\| id:ebi-a-GCST90001433 | Inverse variance weighted | 2 | -0.098 | 0.238 | 0.680 | 0.907 | 0.569 | 1.445 |
| Switched memory B cell %lymphocyte \|\| id:ebi-a-GCST90001434 | Inverse variance weighted | 3 | -0.053 | 0.314 | 0.867 | 0.949 | 0.513 | 1.754 |
| Plasma Blast-Plasma Cell %lymphocyte \|\| id:ebi-a-GCST90001435 | Wald ratio | 1 | 0.163 | 0.326 | 0.616 | 1.177 | 0.622 | 2.229 |
| Memory B cell %lymphocyte \|\| id:ebi-a-GCST90001436 | Inverse variance weighted | 2 | -0.187 | 0.270 | 0.490 | 0.830 | 0.488 | 1.409 |
| Naive-mature B cell %lymphocyte \|\| id:ebi-a-GCST90001437 | Wald ratio | 1 | 0.081 | 0.113 | 0.475 | 1.084 | 0.869 | 1.353 |
| IgD+ CD24+ B cell %lymphocyte \|\| id:ebi-a-GCST90001439 | Inverse variance weighted | 2 | -0.107 | 0.257 | 0.678 | 0.899 | 0.543 | 1.488 |
| IgD+ CD24- B cell %lymphocyte \|\| id:ebi-a-GCST90001441 | Wald ratio | 1 | 0.084 | 0.117 | 0.475 | 1.087 | 0.864 | 1.368 |
| CD24+ CD27+ B cell %lymphocyte \|\| id:ebi-a-GCST90001442 | Inverse variance weighted | 3 | -0.171 | 0.212 | 0.418 | 0.842 | 0.556 | 1.275 |
| IgD- CD38- B cell Absolute Count \|\| id:ebi-a-GCST90001446 | Wald ratio | 1 | 0.129 | 0.216 | 0.553 | 1.137 | 0.744 | 1.738 |
| CD62L- monocyte Absolute Count \|\| id:ebi-a-GCST90001450 | Inverse variance weighted | 3 | 0.004 | 0.157 | 0.982 | 1.004 | 0.738 | 1.365 |
| CD62L- monocyte %monocyte \|\| id:ebi-a-GCST90001451 | Inverse variance weighted | 4 | 0.088 | 0.109 | 0.423 | 1.092 | 0.881 | 1.353 |
| CD11c+ CD62L- monocyte Absolute Count \|\| id:ebi-a-GCST90001452 | Wald ratio | 1 | -0.709 | 0.371 | 0.056 | 0.492 | 0.238 | 1.020 |
| CD11c+ HLA DR++ monocyte %monocyte \|\| id:ebi-a-GCST90001457 | Wald ratio | 1 | -0.119 | 0.268 | 0.657 | 0.888 | 0.525 | 1.502 |
| Myeloid Dendritic Cell Absolute Count \|\| id:ebi-a-GCST90001458 | Inverse variance weighted | 3 | 0.163 | 0.119 | 0.172 | 1.177 | 0.932 | 1.487 |
| Myeloid Dendritic Cell %Dendritic Cell \|\| id:ebi-a-GCST90001459 | Inverse variance weighted | 2 | 0.224 | 0.103 | 0.030 | 1.251 | 1.022 | 1.531 |
| Plasmacytoid Dendritic Cell Absolute Count \|\| id:ebi-a-GCST90001460 | Inverse variance weighted | 2 | -0.132 | 0.192 | 0.491 | 0.876 | 0.602 | 1.276 |
| Dendritic Cell Absolute Count \|\| id:ebi-a-GCST90001461 | Inverse variance weighted | 3 | 0.154 | 0.126 | 0.222 | 1.166 | 0.911 | 1.493 |
| CD62L- Dendritic Cell Absolute Count \|\| id:ebi-a-GCST90001462 | Inverse variance weighted | 5 | 0.119 | 0.084 | 0.158 | 1.126 | 0.955 | 1.328 |
| CD62L- Dendritic Cell %Dendritic Cell \|\| id:ebi-a-GCST90001463 | Inverse variance weighted | 4 | 0.120 | 0.080 | 0.134 | 1.128 | 0.964 | 1.319 |
| CD86+ myeloid Dendritic Cell Absolute Count \|\| id:ebi-a-GCST90001464 | Inverse variance weighted | 5 | 0.118 | 0.078 | 0.130 | 1.125 | 0.966 | 1.310 |
| CD86+ myeloid Dendritic Cell %Dendritic Cell \|\| id:ebi-a-GCST90001465 | Inverse variance weighted | 4 | 0.135 | 0.078 | 0.082 | 1.145 | 0.983 | 1.333 |
| CD86+ plasmacytoid Dendritic Cell %Dendritic Cell \|\| id:ebi-a-GCST90001467 | Wald ratio | 1 | -0.468 | 0.246 | 0.057 | 0.626 | 0.387 | 1.014 |
| CD62L- myeloid Dendritic Cell Absolute Count \|\| id:ebi-a-GCST90001468 | Inverse variance weighted | 6 | 0.125 | 0.073 | 0.087 | 1.133 | 0.982 | 1.307 |
| CD62L- myeloid Dendritic Cell %Dendritic Cell \|\| id:ebi-a-GCST90001469 | Inverse variance weighted | 6 | 0.119 | 0.072 | 0.098 | 1.127 | 0.978 | 1.298 |
| CD62L- plasmacytoid Dendritic Cell Absolute Count \|\| id:ebi-a-GCST90001470 | Wald ratio | 1 | 0.089 | 0.173 | 0.607 | 1.093 | 0.779 | 1.532 |
| CD62L- plasmacytoid Dendritic Cell %Dendritic Cell \|\| id:ebi-a-GCST90001471 | Inverse variance weighted | 2 | -0.089 | 0.240 | 0.710 | 0.915 | 0.571 | 1.464 |
| CD62L- CD86+ myeloid Dendritic Cell Absolute Count \|\| id:ebi-a-GCST90001472 | Inverse variance weighted | 5 | 0.105 | 0.068 | 0.121 | 1.111 | 0.973 | 1.269 |
| CD62L- CD86+ myeloid Dendritic Cell %Dendritic Cell \|\| id:ebi-a-GCST90001473 | Inverse variance weighted | 5 | 0.110 | 0.065 | 0.093 | 1.116 | 0.982 | 1.269 |
| Plasmacytoid Dendritic Cell %Dendritic Cell \|\| id:ebi-a-GCST90001474 | Inverse variance weighted | 2 | -0.258 | 0.104 | 0.013 | 0.772 | 0.630 | 0.946 |
| HLA DR++ monocyte %monocyte \|\| id:ebi-a-GCST90001475 | Inverse variance weighted | 4 | -0.014 | 0.111 | 0.898 | 0.986 | 0.793 | 1.225 |
| HLA DR++ monocyte %leukocyte \|\| id:ebi-a-GCST90001476 | Inverse variance weighted | 2 | -0.165 | 0.151 | 0.273 | 0.848 | 0.631 | 1.139 |
| HLA DR++ monocyte Absolute Count \|\| id:ebi-a-GCST90001477 | Wald ratio | 1 | -0.102 | 0.211 | 0.628 | 0.903 | 0.598 | 1.364 |
| CD4 regulatory T cell %CD4+ T cell \|\| id:ebi-a-GCST90001478 | Inverse variance weighted | 2 | -0.230 | 0.244 | 0.348 | 0.795 | 0.492 | 1.284 |
| CD4 regulatory T cell %T cell \|\| id:ebi-a-GCST90001479 | Wald ratio | 1 | -0.292 | 0.393 | 0.457 | 0.747 | 0.346 | 1.613 |
| Resting CD4 regulatory T cell Absolute Count \|\| id:ebi-a-GCST90001480 | Inverse variance weighted | 4 | 0.006 | 0.077 | 0.936 | 1.006 | 0.865 | 1.170 |
| Resting CD4 regulatory T cell %CD4 regulatory T cell \|\| id:ebi-a-GCST90001481 | Inverse variance weighted | 11 | -0.022 | 0.037 | 0.552 | 0.978 | 0.909 | 1.052 |
| Resting CD4 regulatory T cell %CD4+ T cell \|\| id:ebi-a-GCST90001482 | Inverse variance weighted | 6 | -0.037 | 0.047 | 0.426 | 0.963 | 0.879 | 1.056 |
| CD39+ resting CD4 regulatory T cell Absolute Count \|\| id:ebi-a-GCST90001483 | Inverse variance weighted | 9 | 0.016 | 0.054 | 0.767 | 1.016 | 0.915 | 1.128 |
| CD39+ resting CD4 regulatory T cell %resting CD4 regulatory T cell \|\| id:ebi-a-GCST90001484 | Inverse variance weighted | 7 | 0.021 | 0.068 | 0.759 | 1.021 | 0.894 | 1.167 |
| CD39+ resting CD4 regulatory T cell %CD4 regulatory T cell \|\| id:ebi-a-GCST90001485 | Inverse variance weighted | 13 | 0.000 | 0.030 | 0.988 | 1.000 | 0.943 | 1.062 |
| Activated CD4 regulatory T cell Absolute Count \|\| id:ebi-a-GCST90001486 | Inverse variance weighted | 2 | 0.189 | 0.115 | 0.101 | 1.208 | 0.964 | 1.514 |
| Activated CD4 regulatory T cell %CD4 regulatory T cell \|\| id:ebi-a-GCST90001487 | Inverse variance weighted | 4 | 0.051 | 0.068 | 0.456 | 1.052 | 0.921 | 1.201 |
| CD39+ activated CD4 regulatory T cell Absolute Count \|\| id:ebi-a-GCST90001489 | Inverse variance weighted | 5 | -0.039 | 0.085 | 0.650 | 0.962 | 0.814 | 1.137 |
| CD39+ activated CD4 regulatory T cell %CD4 regulatory T cell \|\| id:ebi-a-GCST90001491 | Inverse variance weighted | 7 | -0.028 | 0.039 | 0.475 | 0.972 | 0.900 | 1.050 |
| Secreting CD4 regulatory T cell Absolute Count \|\| id:ebi-a-GCST90001492 | Inverse variance weighted | 4 | 0.054 | 0.054 | 0.311 | 1.056 | 0.950 | 1.173 |
| Secreting CD4 regulatory T cell %CD4 regulatory T cell \|\| id:ebi-a-GCST90001493 | Inverse variance weighted | 6 | 0.014 | 0.040 | 0.734 | 1.014 | 0.937 | 1.097 |
| Secreting CD4 regulatory T cell %CD4+ T cell \|\| id:ebi-a-GCST90001494 | Inverse variance weighted | 7 | 0.028 | 0.038 | 0.462 | 1.028 | 0.955 | 1.108 |
| CD39+ secreting CD4 regulatory T cell Absolute Count \|\| id:ebi-a-GCST90001495 | Inverse variance weighted | 6 | -0.015 | 0.038 | 0.686 | 0.985 | 0.915 | 1.060 |
| CD39+ secreting CD4 regulatory T cell %secreting CD4 regulatory T cell \|\| id:ebi-a-GCST90001496 | Inverse variance weighted | 8 | -0.020 | 0.034 | 0.571 | 0.981 | 0.917 | 1.049 |
| CD39+ secreting CD4 regulatory T cell %CD4 regulatory T cell \|\| id:ebi-a-GCST90001497 | Inverse variance weighted | 8 | -0.026 | 0.035 | 0.463 | 0.975 | 0.910 | 1.044 |
| Activated & resting CD4 regulatory T cell Absolute Count \|\| id:ebi-a-GCST90001498 | Wald ratio | 1 | -0.131 | 0.279 | 0.639 | 0.877 | 0.508 | 1.515 |
| Activated & resting CD4 regulatory T cell %CD4 regulatory T cell \|\| id:ebi-a-GCST90001499 | Inverse variance weighted | 6 | -0.013 | 0.039 | 0.736 | 0.987 | 0.914 | 1.066 |
| Activated & resting CD4 regulatory T cell %CD4+ T cell \|\| id:ebi-a-GCST90001500 | Inverse variance weighted | 3 | -0.016 | 0.074 | 0.831 | 0.984 | 0.852 | 1.137 |
| Activated & secreting CD4 regulatory T cell Absolute Count \|\| id:ebi-a-GCST90001501 | Inverse variance weighted | 4 | 0.056 | 0.054 | 0.296 | 1.058 | 0.952 | 1.176 |
| Activated & secreting CD4 regulatory T cell %CD4 regulatory T cell \|\| id:ebi-a-GCST90001502 | Inverse variance weighted | 8 | 0.027 | 0.035 | 0.440 | 1.028 | 0.959 | 1.101 |
| Activated & secreting CD4 regulatory T cell %CD4+ T cell \|\| id:ebi-a-GCST90001503 | Inverse variance weighted | 9 | 0.029 | 0.032 | 0.361 | 1.030 | 0.967 | 1.096 |
| CD25++ CD4+ T cell Absolute Count \|\| id:ebi-a-GCST90001504 | Wald ratio | 1 | 0.091 | 0.230 | 0.691 | 1.096 | 0.698 | 1.720 |
| CD25++ CD4+ T cell %CD4+ T cell \|\| id:ebi-a-GCST90001505 | Inverse variance weighted | 2 | 0.047 | 0.163 | 0.774 | 1.048 | 0.761 | 1.442 |
| CD25++ CD4+ T cell %T cell \|\| id:ebi-a-GCST90001506 | Wald ratio | 1 | 0.082 | 0.206 | 0.691 | 1.085 | 0.724 | 1.627 |
| CD25++ CD45RA+ CD4 not regulatory T cell Absolute Count \|\| id:ebi-a-GCST90001507 | Inverse variance weighted | 3 | -0.307 | 0.104 | 0.003 | 0.736 | 0.600 | 0.902 |
| CD25++ CD45RA+ CD4 not regulatory T cell %CD4+ T cell \|\| id:ebi-a-GCST90001508 | Inverse variance weighted | 5 | -0.143 | 0.074 | 0.055 | 0.867 | 0.749 | 1.003 |
| CD25++ CD45RA+ CD4 not regulatory T cell %T cell \|\| id:ebi-a-GCST90001509 | Inverse variance weighted | 5 | -0.150 | 0.078 | 0.056 | 0.861 | 0.738 | 1.004 |
| CD25++ CD45RA- CD4 not regulatory T cell Absolute Count \|\| id:ebi-a-GCST90001510 | Inverse variance weighted | 2 | 0.082 | 0.136 | 0.544 | 1.086 | 0.832 | 1.416 |
| CD25++ CD45RA- CD4 not regulatory T cell %CD4+ T cell \|\| id:ebi-a-GCST90001511 | Inverse variance weighted | 3 | 0.002 | 0.075 | 0.978 | 1.002 | 0.865 | 1.160 |
| CD25++ CD45RA- CD4 not regulatory T cell %T cell \|\| id:ebi-a-GCST90001512 | Inverse variance weighted | 3 | 0.016 | 0.066 | 0.808 | 1.016 | 0.893 | 1.156 |
| Hematopoietic Stem Cell Absolute Count \|\| id:ebi-a-GCST90001514 | Inverse variance weighted | 2 | 0.064 | 0.087 | 0.465 | 1.066 | 0.898 | 1.264 |
| Immature Myeloid-Derived Suppressor Cells Absolute Count \|\| id:ebi-a-GCST90001515 | Inverse variance weighted | 2 | 0.125 | 0.166 | 0.451 | 1.133 | 0.819 | 1.568 |
| Immature Myeloid-Derived Suppressor Cells %CD33dim HLA DR- CD66b- \|\| id:ebi-a-GCST90001516 | Inverse variance weighted | 2 | 0.103 | 0.189 | 0.586 | 1.108 | 0.765 | 1.606 |
| CD33+ HLA DR+ Absolute Count \|\| id:ebi-a-GCST90001517 | Wald ratio | 1 | 0.211 | 0.118 | 0.073 | 1.235 | 0.980 | 1.557 |
| CD33+ HLA DR+ CD14- Absolute Count \|\| id:ebi-a-GCST90001518 | Wald ratio | 1 | 0.207 | 0.116 | 0.073 | 1.230 | 0.981 | 1.544 |
| CD33+ HLA DR+ CD14- %CD33+ HLA DR+ \|\| id:ebi-a-GCST90001519 | Wald ratio | 1 | 0.236 | 0.139 | 0.089 | 1.266 | 0.964 | 1.663 |
| CD33+ HLA DR+ CD14dim Absolute Count \|\| id:ebi-a-GCST90001520 | Inverse variance weighted | 2 | 0.205 | 0.120 | 0.086 | 1.228 | 0.971 | 1.553 |
| CD33+ HLA DR+ CD14dim %CD33+ HLA DR+ \|\| id:ebi-a-GCST90001521 | Wald ratio | 1 | 0.346 | 0.185 | 0.062 | 1.413 | 0.983 | 2.030 |
| CD33- HLA DR- Absolute Count \|\| id:ebi-a-GCST90001522 | Wald ratio | 1 | -0.120 | 0.226 | 0.597 | 0.887 | 0.569 | 1.383 |
| CD33- HLA DR+ Absolute Count \|\| id:ebi-a-GCST90001523 | Wald ratio | 1 | -0.274 | 0.180 | 0.128 | 0.760 | 0.534 | 1.082 |
| Granulocytic Myeloid-Derived Suppressor Cells Absolute Count \|\| id:ebi-a-GCST90001524 | Inverse variance weighted | 2 | 0.188 | 0.182 | 0.301 | 1.207 | 0.845 | 1.725 |
| CD33dim HLA DR+ CD11b+ Absolute Count \|\| id:ebi-a-GCST90001525 | Wald ratio | 1 | -0.064 | 0.160 | 0.690 | 0.938 | 0.685 | 1.284 |
| CD33dim HLA DR+ CD11b+ %CD33dim HLA DR+ \|\| id:ebi-a-GCST90001526 | Inverse variance weighted | 3 | -0.080 | 0.061 | 0.191 | 0.923 | 0.819 | 1.041 |
| CD33dim HLA DR+ CD11b- Absolute Count \|\| id:ebi-a-GCST90001527 | Wald ratio | 1 | 0.112 | 0.147 | 0.445 | 1.119 | 0.839 | 1.492 |
| CD33dim HLA DR+ CD11b- %CD33dim HLA DR+ \|\| id:ebi-a-GCST90001528 | Inverse variance weighted | 3 | 0.080 | 0.061 | 0.193 | 1.083 | 0.960 | 1.221 |
| Monocytic Myeloid-Derived Suppressor Cells Absolute Count \|\| id:ebi-a-GCST90001530 | Inverse variance weighted | 5 | 0.101 | 0.067 | 0.127 | 1.107 | 0.971 | 1.261 |
| CD33dim HLA DR- Absolute Count \|\| id:ebi-a-GCST90001531 | Inverse variance weighted | 3 | 0.076 | 0.036 | 0.036 | 1.079 | 1.005 | 1.159 |
| Basophil Absolute Count \|\| id:ebi-a-GCST90001532 | Inverse variance weighted | 3 | 0.076 | 0.036 | 0.036 | 1.079 | 1.005 | 1.159 |
| Basophil %CD33dim HLA DR- CD66b- \|\| id:ebi-a-GCST90001533 | Inverse variance weighted | 3 | 0.143 | 0.073 | 0.050 | 1.154 | 1.000 | 1.330 |
| CD45RA- CD4+ T cell Absolute Count \|\| id:ebi-a-GCST90001534 | Inverse variance weighted | 2 | 0.311 | 0.127 | 0.015 | 1.365 | 1.063 | 1.752 |
| CD45RA- CD4+ T cell %CD4+ T cell \|\| id:ebi-a-GCST90001535 | Inverse variance weighted | 4 | 0.090 | 0.083 | 0.277 | 1.095 | 0.930 | 1.288 |
| CD45RA- CD4+ T cell %T cell \|\| id:ebi-a-GCST90001536 | Inverse variance weighted | 2 | 0.326 | 0.137 | 0.018 | 1.386 | 1.059 | 1.814 |
| Central Memory CD4+ T cell Absolute Count \|\| id:ebi-a-GCST90001537 | Wald ratio | 1 | 0.309 | 0.265 | 0.244 | 1.362 | 0.810 | 2.291 |
| Central Memory CD4+ T cell %CD4+ T cell \|\| id:ebi-a-GCST90001538 | Wald ratio | 1 | 0.114 | 0.243 | 0.639 | 1.121 | 0.696 | 1.806 |
| Naive CD4+ T cell Absolute Count \|\| id:ebi-a-GCST90001540 | Inverse variance weighted | 2 | -0.104 | 0.157 | 0.508 | 0.901 | 0.662 | 1.226 |
| Naive CD4+ T cell %CD4+ T cell \|\| id:ebi-a-GCST90001541 | Inverse variance weighted | 4 | -0.285 | 0.125 | 0.022 | 0.752 | 0.589 | 0.960 |
| Effector Memory CD4+ T cell Absolute Count \|\| id:ebi-a-GCST90001542 | Wald ratio | 1 | 0.327 | 0.150 | 0.029 | 1.386 | 1.034 | 1.859 |
| Effector Memory CD4+ T cell %CD4+ T cell \|\| id:ebi-a-GCST90001543 | Inverse variance weighted | 2 | 0.397 | 0.157 | 0.011 | 1.487 | 1.094 | 2.022 |
| Effector Memory CD4+ T cell %T cell \|\| id:ebi-a-GCST90001544 | Inverse variance weighted | 4 | 0.222 | 0.151 | 0.142 | 1.249 | 0.929 | 1.679 |
| Terminally Differentiated CD4+ T cell Absolute Count \|\| id:ebi-a-GCST90001545 | Inverse variance weighted | 2 | 0.011 | 0.235 | 0.962 | 1.011 | 0.638 | 1.603 |
| Terminally Differentiated CD4+ T cell %CD4+ T cell \|\| id:ebi-a-GCST90001546 | Inverse variance weighted | 5 | 0.026 | 0.116 | 0.825 | 1.026 | 0.817 | 1.289 |
| Terminally Differentiated CD4+ T cell %T cell \|\| id:ebi-a-GCST90001547 | Inverse variance weighted | 6 | 0.044 | 0.082 | 0.589 | 1.045 | 0.890 | 1.229 |
| Central Memory CD8+ T cell Absolute Count \|\| id:ebi-a-GCST90001548 | Wald ratio | 1 | 0.121 | 0.263 | 0.645 | 1.128 | 0.674 | 1.889 |
| Naive CD8+ T cell Absolute Count \|\| id:ebi-a-GCST90001551 | Inverse variance weighted | 2 | -0.032 | 0.292 | 0.912 | 0.968 | 0.546 | 1.718 |
| Naive CD8+ T cell %CD8+ T cell \|\| id:ebi-a-GCST90001552 | Wald ratio | 1 | -0.132 | 0.402 | 0.743 | 0.876 | 0.399 | 1.928 |
| Naive CD8+ T cell %T cell \|\| id:ebi-a-GCST90001553 | Inverse variance weighted | 3 | 0.013 | 0.011 | 0.230 | 1.013 | 0.992 | 1.035 |
| Effector Memory CD8+ T cell Absolute Count \|\| id:ebi-a-GCST90001554 | Inverse variance weighted | 2 | 0.010 | 0.090 | 0.912 | 1.010 | 0.847 | 1.205 |
| Effector Memory CD8+ T cell %CD8+ T cell \|\| id:ebi-a-GCST90001555 | Inverse variance weighted | 4 | -0.005 | 0.071 | 0.946 | 0.995 | 0.865 | 1.145 |
| Effector Memory CD8+ T cell %T cell \|\| id:ebi-a-GCST90001556 | Inverse variance weighted | 2 | 0.023 | 0.090 | 0.798 | 1.023 | 0.858 | 1.221 |
| Terminally Differentiated CD8+ T cell Absolute Count \|\| id:ebi-a-GCST90001557 | Wald ratio | 1 | -0.407 | 0.336 | 0.226 | 0.665 | 0.344 | 1.286 |
| Terminally Differentiated CD8+ T cell %CD8+ T cell \|\| id:ebi-a-GCST90001558 | Inverse variance weighted | 3 | -0.275 | 0.132 | 0.037 | 0.760 | 0.587 | 0.983 |
| Terminally Differentiated CD8+ T cell %T cell \|\| id:ebi-a-GCST90001559 | Inverse variance weighted | 2 | -0.403 | 0.161 | 0.013 | 0.669 | 0.487 | 0.917 |
| CD45RA+ CD8+ T cell Absolute Count \|\| id:ebi-a-GCST90001560 | Inverse variance weighted | 3 | -0.305 | 0.191 | 0.111 | 0.737 | 0.507 | 1.072 |
| CD45RA+ CD8+ T cell %CD8+ T cell \|\| id:ebi-a-GCST90001561 | Inverse variance weighted | 5 | 0.003 | 0.031 | 0.932 | 1.003 | 0.944 | 1.065 |
| CD45RA+ CD8+ T cell %T cell \|\| id:ebi-a-GCST90001562 | Inverse variance weighted | 3 | -0.316 | 0.153 | 0.038 | 0.729 | 0.540 | 0.983 |
| Naive CD4-CD8- T cell %T cell \|\| id:ebi-a-GCST90001568 | Wald ratio | 1 | -0.139 | 0.382 | 0.717 | 0.871 | 0.411 | 1.842 |
| Effector Memory CD4-CD8- T cell Absolute Count \|\| id:ebi-a-GCST90001569 | Inverse variance weighted | 4 | -0.021 | 0.129 | 0.872 | 0.979 | 0.761 | 1.261 |
| Effector Memory CD4-CD8- T cell %CD4-CD8- T cell \|\| id:ebi-a-GCST90001570 | Inverse variance weighted | 4 | -0.055 | 0.110 | 0.618 | 0.946 | 0.762 | 1.175 |
| Effector Memory CD4-CD8- T cell %T cell \|\| id:ebi-a-GCST90001571 | Inverse variance weighted | 3 | -0.290 | 0.201 | 0.150 | 0.748 | 0.504 | 1.110 |
| Terminally Differentiated CD4-CD8- T cell Absolute Count \|\| id:ebi-a-GCST90001572 | Inverse variance weighted | 2 | 0.092 | 0.364 | 0.800 | 1.097 | 0.537 | 2.240 |
| Terminally Differentiated CD4-CD8- T cell %CD4-CD8- T cell \|\| id:ebi-a-GCST90001573 | Inverse variance weighted | 2 | -0.138 | 0.169 | 0.414 | 0.871 | 0.626 | 1.212 |
| Terminally Differentiated CD4-CD8- T cell %T cell \|\| id:ebi-a-GCST90001574 | Inverse variance weighted | 4 | -0.074 | 0.164 | 0.654 | 0.929 | 0.673 | 1.282 |
| Transitional B cell Absolute Count \|\| id:ebi-a-GCST90001577 | Wald ratio | 1 | -0.114 | 0.344 | 0.742 | 0.893 | 0.455 | 1.753 |
| CD14- CD16+ monocyte Absolute Count \|\| id:ebi-a-GCST90001579 | Wald ratio | 1 | -0.365 | 0.190 | 0.055 | 0.694 | 0.478 | 1.008 |
| CD14+ CD16+ monocyte Absolute Count \|\| id:ebi-a-GCST90001580 | Inverse variance weighted | 3 | -0.183 | 0.095 | 0.055 | 0.833 | 0.691 | 1.004 |
| CD14- CD16- Absolute Count \|\| id:ebi-a-GCST90001581 | Inverse variance weighted | 4 | 0.077 | 0.170 | 0.652 | 1.080 | 0.773 | 1.508 |
| CD14+ CD16- monocyte Absolute Count \|\| id:ebi-a-GCST90001582 | Inverse variance weighted | 3 | -0.047 | 0.186 | 0.802 | 0.954 | 0.663 | 1.375 |
| Monocyte Absolute Count \|\| id:ebi-a-GCST90001583 | Wald ratio | 1 | -0.089 | 0.306 | 0.770 | 0.915 | 0.503 | 1.665 |
| CD14- CD16+ monocyte %monocyte \|\| id:ebi-a-GCST90001584 | Inverse variance weighted | 2 | -0.178 | 0.290 | 0.541 | 0.837 | 0.474 | 1.479 |
| CD14+ CD16+ monocyte %monocyte \|\| id:ebi-a-GCST90001585 | Inverse variance weighted | 3 | -0.173 | 0.088 | 0.050 | 0.841 | 0.707 | 1.000 |
| CD14+ CD16- monocyte %monocyte \|\| id:ebi-a-GCST90001586 | Inverse variance weighted | 2 | 0.337 | 0.155 | 0.030 | 1.401 | 1.034 | 1.899 |
| CD16+ monocyte %monocyte \|\| id:ebi-a-GCST90001587 | Inverse variance weighted | 2 | -0.343 | 0.157 | 0.029 | 0.710 | 0.521 | 0.966 |
| T/B cell \|\| id:ebi-a-GCST90001588 | Inverse variance weighted | 2 | 0.212 | 0.243 | 0.384 | 1.236 | 0.767 | 1.991 |
| CD4+/CD8+ T cell \|\| id:ebi-a-GCST90001589 | Wald ratio | 1 | 0.229 | 0.467 | 0.624 | 1.257 | 0.504 | 3.138 |
| CD4+ T cell Absolute Count \|\| id:ebi-a-GCST90001590 | Wald ratio | 1 | 0.270 | 0.232 | 0.244 | 1.310 | 0.831 | 2.066 |
| CD8+ T cell Absolute Count \|\| id:ebi-a-GCST90001592 | Inverse variance weighted | 2 | -0.097 | 0.259 | 0.708 | 0.908 | 0.546 | 1.508 |
| CD8+ T cell %T cell \|\| id:ebi-a-GCST90001593 | Wald ratio | 1 | -0.225 | 0.460 | 0.624 | 0.798 | 0.324 | 1.965 |
| CD4+CD8+ T cell Absolute Count \|\| id:ebi-a-GCST90001594 | Inverse variance weighted | 2 | -0.084 | 0.152 | 0.582 | 0.920 | 0.683 | 1.239 |
| CD8dim T cell Absolute Count \|\| id:ebi-a-GCST90001596 | Inverse variance weighted | 2 | -0.005 | 0.250 | 0.983 | 0.995 | 0.609 | 1.625 |
| CD8dim T cell %T cell \|\| id:ebi-a-GCST90001597 | Inverse variance weighted | 2 | -0.005 | 0.215 | 0.982 | 0.995 | 0.653 | 1.517 |
| CD4-CD8- T cell Absolute Count \|\| id:ebi-a-GCST90001598 | Inverse variance weighted | 2 | -0.085 | 0.211 | 0.686 | 0.918 | 0.607 | 1.390 |
| CD4-CD8- T cell %T cell \|\| id:ebi-a-GCST90001599 | Inverse variance weighted | 3 | -0.140 | 0.174 | 0.419 | 0.869 | 0.618 | 1.222 |
| Lymphocyte Absolute Count \|\| id:ebi-a-GCST90001601 | Inverse variance weighted | 2 | 0.027 | 0.264 | 0.919 | 1.027 | 0.612 | 1.722 |
| Lymphocyte %leukocyte \|\| id:ebi-a-GCST90001602 | Wald ratio | 1 | -0.231 | 0.427 | 0.589 | 0.794 | 0.343 | 1.834 |
| T cell Absolute Count \|\| id:ebi-a-GCST90001603 | Inverse variance weighted | 2 | 0.011 | 0.261 | 0.968 | 1.011 | 0.606 | 1.685 |
| T cell %lymphocyte \|\| id:ebi-a-GCST90001604 | Inverse variance weighted | 2 | -0.178 | 0.272 | 0.513 | 0.837 | 0.491 | 1.427 |
| CD4+ T cell %leukocyte \|\| id:ebi-a-GCST90001606 | Wald ratio | 1 | -0.285 | 0.421 | 0.499 | 0.752 | 0.330 | 1.716 |
| CD8+ T cell %leukocyte \|\| id:ebi-a-GCST90001607 | Wald ratio | 1 | 0.130 | 0.251 | 0.603 | 1.139 | 0.697 | 1.863 |
| CD4+CD8+ T cell %leukocyte \|\| id:ebi-a-GCST90001608 | Wald ratio | 1 | -0.065 | 0.150 | 0.666 | 0.937 | 0.698 | 1.258 |
| CD4+ CD8dim T cell Absolute Count \|\| id:ebi-a-GCST90001609 | Inverse variance weighted | 2 | 0.244 | 0.132 | 0.065 | 1.276 | 0.985 | 1.653 |
| CD4+ CD8dim T cell %lymphocyte \|\| id:ebi-a-GCST90001610 | Inverse variance weighted | 2 | 0.224 | 0.119 | 0.060 | 1.252 | 0.990 | 1.582 |
| CD4+ CD8dim T cell %leukocyte \|\| id:ebi-a-GCST90001611 | Inverse variance weighted | 2 | 0.227 | 0.122 | 0.062 | 1.255 | 0.989 | 1.594 |
| CD8dim T cell %leukocyte \|\| id:ebi-a-GCST90001612 | Inverse variance weighted | 2 | -0.009 | 0.227 | 0.970 | 0.992 | 0.635 | 1.548 |
| CD4-CD8- T cell %leukocyte \|\| id:ebi-a-GCST90001613 | Inverse variance weighted | 4 | -0.066 | 0.158 | 0.675 | 0.936 | 0.687 | 1.275 |
| CD8+ and CD8dim T cell %leukocyte \|\| id:ebi-a-GCST90001614 | Inverse variance weighted | 2 | -0.372 | 0.253 | 0.141 | 0.690 | 0.420 | 1.131 |
| TCRgd T cell Absolute Count \|\| id:ebi-a-GCST90001615 | Wald ratio | 1 | 0.226 | 0.267 | 0.397 | 1.254 | 0.743 | 2.114 |
| HLA DR+ T cell Absolute Count \|\| id:ebi-a-GCST90001618 | Inverse variance weighted | 4 | 0.010 | 0.093 | 0.917 | 1.010 | 0.841 | 1.212 |
| HLA DR+ T cell%T cell \|\| id:ebi-a-GCST90001619 | Inverse variance weighted | 4 | -0.019 | 0.089 | 0.834 | 0.982 | 0.824 | 1.169 |
| HLA DR+ T cell%lymphocyte \|\| id:ebi-a-GCST90001620 | Inverse variance weighted | 4 | -0.056 | 0.088 | 0.524 | 0.945 | 0.795 | 1.124 |
| Natural Killer T Absolute Count \|\| id:ebi-a-GCST90001621 | Inverse variance weighted | 5 | 0.279 | 0.179 | 0.119 | 1.322 | 0.931 | 1.878 |
| Natural Killer T %T cell \|\| id:ebi-a-GCST90001622 | Inverse variance weighted | 7 | 0.204 | 0.171 | 0.232 | 1.226 | 0.878 | 1.714 |
| Natural Killer T %lymphocyte \|\| id:ebi-a-GCST90001623 | Inverse variance weighted | 8 | -0.012 | 0.126 | 0.923 | 0.988 | 0.772 | 1.264 |
| HLA DR+ CD4+ T cell Absolute Count \|\| id:ebi-a-GCST90001624 | Inverse variance weighted | 2 | -0.005 | 0.140 | 0.974 | 0.995 | 0.756 | 1.310 |
| HLA DR+ CD4+ T cell %T cell \|\| id:ebi-a-GCST90001625 | Wald ratio | 1 | -0.219 | 0.173 | 0.205 | 0.803 | 0.572 | 1.128 |
| HLA DR+ CD4+ T cell %lymphocyte \|\| id:ebi-a-GCST90001626 | Inverse variance weighted | 2 | -0.011 | 0.137 | 0.935 | 0.989 | 0.756 | 1.294 |
| HLA DR+ CD8+ T cell Absolute Count \|\| id:ebi-a-GCST90001627 | Inverse variance weighted | 4 | -0.060 | 0.091 | 0.506 | 0.941 | 0.788 | 1.124 |
| HLA DR+ CD8+ T cell %T cell \|\| id:ebi-a-GCST90001628 | Inverse variance weighted | 4 | -0.034 | 0.109 | 0.757 | 0.967 | 0.781 | 1.197 |
| HLA DR+ CD8+ T cell %lymphocyte \|\| id:ebi-a-GCST90001629 | Inverse variance weighted | 3 | -0.079 | 0.094 | 0.402 | 0.924 | 0.769 | 1.111 |
| CD8+ Natural Killer T Absolute Count \|\| id:ebi-a-GCST90001630 | Inverse variance weighted | 2 | -0.102 | 0.333 | 0.760 | 0.903 | 0.470 | 1.735 |
| CD8+ Natural Killer T %T cell \|\| id:ebi-a-GCST90001631 | Wald ratio | 1 | 0.890 | 0.480 | 0.064 | 2.436 | 0.950 | 6.245 |
| CD8+ Natural Killer T %lymphocyte \|\| id:ebi-a-GCST90001632 | Wald ratio | 1 | 0.438 | 0.447 | 0.327 | 1.549 | 0.645 | 3.717 |
| CD8dim Natural Killer T Absolute Count \|\| id:ebi-a-GCST90001633 | Inverse variance weighted | 6 | 0.006 | 0.115 | 0.957 | 1.006 | 0.804 | 1.260 |
| CD8dim Natural Killer T %T cell \|\| id:ebi-a-GCST90001634 | Inverse variance weighted | 6 | -0.005 | 0.107 | 0.965 | 0.995 | 0.808 | 1.227 |
| CD8dim Natural Killer T %lymphocyte \|\| id:ebi-a-GCST90001635 | Inverse variance weighted | 7 | 0.016 | 0.103 | 0.879 | 1.016 | 0.830 | 1.243 |
| CD4-CD8- Natural Killer T Absolute Count \|\| id:ebi-a-GCST90001636 | Wald ratio | 1 | -0.359 | 0.253 | 0.156 | 0.698 | 0.425 | 1.146 |
| CD4-CD8- Natural Killer T %T cell \|\| id:ebi-a-GCST90001637 | Inverse variance weighted | 6 | -0.077 | 0.117 | 0.512 | 0.926 | 0.736 | 1.165 |
| CD4-CD8- Natural Killer T %lymphocyte \|\| id:ebi-a-GCST90001638 | Inverse variance weighted | 6 | -0.079 | 0.122 | 0.517 | 0.924 | 0.728 | 1.173 |
| CD3- lymphocyte Absolute Count \|\| id:ebi-a-GCST90001639 | Wald ratio | 1 | 0.244 | 0.205 | 0.235 | 1.276 | 0.854 | 1.907 |
| CD3- lymphocyte %leukocyte \|\| id:ebi-a-GCST90001641 | Wald ratio | 1 | 0.553 | 0.329 | 0.092 | 1.739 | 0.913 | 3.312 |
| B cell Absolute Count \|\| id:ebi-a-GCST90001642 | Wald ratio | 1 | 0.646 | 0.337 | 0.055 | 1.908 | 0.986 | 3.694 |
| B cell %CD3- lymphocyte \|\| id:ebi-a-GCST90001643 | Inverse variance weighted | 3 | 0.043 | 0.119 | 0.721 | 1.044 | 0.826 | 1.319 |
| B cell %lymphocyte \|\| id:ebi-a-GCST90001644 | Inverse variance weighted | 2 | -0.099 | 0.250 | 0.694 | 0.906 | 0.555 | 1.481 |
| Natural Killer Absolute Count \|\| id:ebi-a-GCST90001645 | Inverse variance weighted | 3 | 0.080 | 0.233 | 0.730 | 1.084 | 0.687 | 1.711 |
| Natural Killer %CD3- lymphocyte \|\| id:ebi-a-GCST90001646 | Inverse variance weighted | 5 | 0.035 | 0.093 | 0.706 | 1.036 | 0.863 | 1.244 |
| Natural Killer %lymphocyte \|\| id:ebi-a-GCST90001647 | Inverse variance weighted | 3 | 0.104 | 0.208 | 0.618 | 1.109 | 0.737 | 1.669 |
| HLA DR+ Natural Killer Absolute Count \|\| id:ebi-a-GCST90001648 | Inverse variance weighted | 4 | -0.113 | 0.085 | 0.181 | 0.893 | 0.756 | 1.054 |
| HLA DR+ Natural Killer %Natural Killer \|\| id:ebi-a-GCST90001649 | Inverse variance weighted | 6 | -0.105 | 0.075 | 0.165 | 0.901 | 0.777 | 1.044 |
| HLA DR+ Natural Killer %CD3- lymphocyte \|\| id:ebi-a-GCST90001650 | Inverse variance weighted | 4 | -0.133 | 0.081 | 0.101 | 0.875 | 0.747 | 1.026 |
| Granulocyte %leukocyte \|\| id:ebi-a-GCST90001652 | Wald ratio | 1 | 0.229 | 0.425 | 0.589 | 1.258 | 0.547 | 2.891 |
| CD28- CD4-CD8- T cell %CD4-CD8- T cell \|\| id:ebi-a-GCST90001653 | Wald ratio | 1 | -0.499 | 0.296 | 0.092 | 0.607 | 0.340 | 1.085 |
| CD28- CD4-CD8- T cell Absolute Count \|\| id:ebi-a-GCST90001654 | Inverse variance weighted | 2 | -0.088 | 0.259 | 0.735 | 0.916 | 0.551 | 1.522 |
| CD28+ CD4-CD8- T cell %T cell \|\| id:ebi-a-GCST90001655 | Wald ratio | 1 | -0.460 | 0.324 | 0.156 | 0.631 | 0.334 | 1.192 |
| CD28+ CD4-CD8- T cell %CD4-CD8- T cell \|\| id:ebi-a-GCST90001656 | Wald ratio | 1 | 0.499 | 0.296 | 0.092 | 1.646 | 0.921 | 2.941 |
| CD39+ CD4+ T cell %T cell \|\| id:ebi-a-GCST90001658 | Inverse variance weighted | 7 | -0.025 | 0.036 | 0.486 | 0.975 | 0.909 | 1.046 |
| CD39+ CD4+ T cell %CD4+ T cell \|\| id:ebi-a-GCST90001659 | Inverse variance weighted | 7 | -0.025 | 0.035 | 0.477 | 0.975 | 0.910 | 1.045 |
| CD39+ CD4+ T cell Absolute Count \|\| id:ebi-a-GCST90001660 | Inverse variance weighted | 6 | -0.058 | 0.066 | 0.381 | 0.944 | 0.829 | 1.074 |
| CD28- CD8dim T cell %T cell \|\| id:ebi-a-GCST90001661 | Inverse variance weighted | 3 | -0.416 | 0.171 | 0.015 | 0.660 | 0.472 | 0.923 |
| CD28- CD8dim T cell %CD8dim T cell \|\| id:ebi-a-GCST90001662 | Wald ratio | 1 | -0.532 | 0.316 | 0.092 | 0.588 | 0.316 | 1.091 |
| CD28- CD8dim T cell Absolute Count \|\| id:ebi-a-GCST90001663 | Inverse variance weighted | 2 | -0.189 | 0.520 | 0.717 | 0.828 | 0.299 | 2.294 |
| CD28+ CD45RA+ CD8dim T cell %T cell \|\| id:ebi-a-GCST90001664 | Inverse variance weighted | 6 | 0.000 | 0.054 | 0.998 | 1.000 | 0.899 | 1.112 |
| CD28+ CD45RA+ CD8dim T cell %CD8dim T cell \|\| id:ebi-a-GCST90001665 | Inverse variance weighted | 5 | -0.060 | 0.054 | 0.268 | 0.942 | 0.847 | 1.047 |
| CD28+ CD45RA+ CD8dim T cell Absolute Count \|\| id:ebi-a-GCST90001666 | Inverse variance weighted | 8 | -0.004 | 0.010 | 0.707 | 0.996 | 0.978 | 1.015 |
| CD28+ CD45RA- CD8dim T cell %T cell \|\| id:ebi-a-GCST90001667 | Inverse variance weighted | 3 | 0.061 | 0.070 | 0.383 | 1.063 | 0.927 | 1.220 |
| CD28+ CD45RA- CD8dim T cell %CD8dim T cell \|\| id:ebi-a-GCST90001668 | Inverse variance weighted | 2 | 0.063 | 0.109 | 0.561 | 1.065 | 0.861 | 1.319 |
| CD28+ CD45RA- CD8dim T cell Absolute Count \|\| id:ebi-a-GCST90001669 | Inverse variance weighted | 3 | 0.097 | 0.083 | 0.245 | 1.101 | 0.936 | 1.296 |
| CD39+ CD8+ T cell %T cell \|\| id:ebi-a-GCST90001670 | Inverse variance weighted | 8 | -0.009 | 0.037 | 0.816 | 0.991 | 0.922 | 1.066 |
| CD39+ CD8+ T cell %CD8+ T cell \|\| id:ebi-a-GCST90001671 | Inverse variance weighted | 6 | 0.009 | 0.086 | 0.915 | 1.009 | 0.852 | 1.195 |
| CD39+ CD8+ T cell Absolute Count \|\| id:ebi-a-GCST90001672 | Inverse variance weighted | 7 | -0.008 | 0.037 | 0.837 | 0.992 | 0.922 | 1.068 |
| CD28- CD127- CD25++ CD8+ T cell Absolute Count \|\| id:ebi-a-GCST90001675 | Wald ratio | 1 | -0.454 | 0.290 | 0.117 | 0.635 | 0.360 | 1.120 |
| CD28- CD25++ CD8+ T cell Absolute Count \|\| id:ebi-a-GCST90001678 | Wald ratio | 1 | -0.322 | 0.289 | 0.265 | 0.725 | 0.412 | 1.276 |
| CD25++ CD8+ T cell %T cell \|\| id:ebi-a-GCST90001679 | Inverse variance weighted | 2 | -0.285 | 0.165 | 0.084 | 0.752 | 0.545 | 1.039 |
| CD25++ CD8+ T cell %CD8+ T cell \|\| id:ebi-a-GCST90001680 | Wald ratio | 1 | -0.329 | 0.130 | 0.011 | 0.720 | 0.558 | 0.928 |
| CD25++ CD8+ T cell Absolute Count \|\| id:ebi-a-GCST90001681 | Inverse variance weighted | 3 | -0.308 | 0.113 | 0.007 | 0.735 | 0.589 | 0.918 |
| CD127- CD8+ T cell Absolute Count \|\| id:ebi-a-GCST90001684 | Wald ratio | 1 | 0.123 | 0.237 | 0.603 | 1.131 | 0.711 | 1.801 |
| CD28- CD8+ T cell %CD8+ T cell \|\| id:ebi-a-GCST90001686 | Inverse variance weighted | 3 | -0.308 | 0.188 | 0.101 | 0.735 | 0.509 | 1.062 |
| CD28- CD8+ T cell Absolute Count \|\| id:ebi-a-GCST90001687 | Inverse variance weighted | 3 | -0.102 | 0.222 | 0.645 | 0.903 | 0.584 | 1.396 |
| CD28+ CD45RA+ CD8+ T cell %T cell \|\| id:ebi-a-GCST90001688 | Inverse variance weighted | 36 | -0.001 | 0.005 | 0.860 | 0.999 | 0.990 | 1.009 |
| CD28+ CD45RA+ CD8+ T cell %CD8+ T cell \|\| id:ebi-a-GCST90001689 | Wald ratio | 1 | 0.012 | 0.146 | 0.935 | 1.012 | 0.760 | 1.348 |
| CD28+ CD45RA+ CD8+ T cell Absolute Count \|\| id:ebi-a-GCST90001690 | Inverse variance weighted | 3 | -0.018 | 0.237 | 0.939 | 0.982 | 0.617 | 1.564 |
| CD28+ CD45RA- CD8+ T cell %T cell \|\| id:ebi-a-GCST90001691 | Inverse variance weighted | 3 | -0.021 | 0.066 | 0.754 | 0.979 | 0.860 | 1.116 |
| CD28+ CD45RA- CD8+ T cell %CD8+ T cell \|\| id:ebi-a-GCST90001692 | Inverse variance weighted | 3 | 0.071 | 0.074 | 0.340 | 1.073 | 0.928 | 1.242 |
| CD28+ CD45RA- CD8+ T cell Absolute Count \|\| id:ebi-a-GCST90001693 | Inverse variance weighted | 2 | 0.013 | 0.059 | 0.820 | 1.014 | 0.903 | 1.138 |
| CD28- CD4-CD8- T cell %T cell \|\| id:ebi-a-GCST90001694 | Inverse variance weighted | 3 | -0.022 | 0.164 | 0.894 | 0.978 | 0.710 | 1.349 |
| CD45RA- CD28- CD8+ T cell Absolute Count \|\| id:ebi-a-GCST90001695 | Inverse variance weighted | 142 | 0.000 | 0.000 | 0.708 | 1.000 | 1.000 | 1.000 |
| CD45RA- CD28- CD8+ T cell %CD8+ T cell \|\| id:ebi-a-GCST90001696 | Inverse variance weighted | 10 | 0.002 | 0.002 | 0.459 | 1.002 | 0.997 | 1.006 |
| CD45RA- CD28- CD8+ T cell %T cell \|\| id:ebi-a-GCST90001697 | Inverse variance weighted | 42 | 0.001 | 0.001 | 0.260 | 1.001 | 0.999 | 1.003 |
| CD45RA+ CD28- CD8+ T cell Absolute Count \|\| id:ebi-a-GCST90001698 | Inverse variance weighted | 425 | 0.000 | 0.000 | 0.369 | 1.000 | 1.000 | 1.000 |
| CD45RA+ CD28- CD8+ T cell %CD8+ T cell \|\| id:ebi-a-GCST90001699 | Inverse variance weighted | 2 | -0.002 | 0.005 | 0.616 | 0.998 | 0.989 | 1.007 |
| CD45RA+ CD28- CD8+ T cell %T cell \|\| id:ebi-a-GCST90001700 | Inverse variance weighted | 62 | 0.000 | 0.001 | 0.624 | 1.000 | 0.999 | 1.001 |
| BAFF-R on CD20- CD38- B cell \|\| id:ebi-a-GCST90001701 | Wald ratio | 1 | 0.017 | 0.144 | 0.906 | 1.017 | 0.767 | 1.349 |
| BAFF-R on CD24+ CD27+ B cell \|\| id:ebi-a-GCST90001702 | Inverse variance weighted | 9 | -0.008 | 0.044 | 0.854 | 0.992 | 0.911 | 1.080 |
| BAFF-R on IgD+ CD24+ B cell \|\| id:ebi-a-GCST90001703 | Inverse variance weighted | 7 | -0.007 | 0.057 | 0.907 | 0.993 | 0.889 | 1.110 |
| BAFF-R on IgD+ CD24- B cell \|\| id:ebi-a-GCST90001704 | Inverse variance weighted | 10 | 0.041 | 0.041 | 0.314 | 1.042 | 0.962 | 1.129 |
| BAFF-R on IgD+ CD38- B cell \|\| id:ebi-a-GCST90001705 | Inverse variance weighted | 9 | 0.040 | 0.042 | 0.344 | 1.041 | 0.958 | 1.130 |
| BAFF-R on IgD+ CD38- naive B cell \|\| id:ebi-a-GCST90001706 | Inverse variance weighted | 8 | 0.040 | 0.041 | 0.325 | 1.041 | 0.961 | 1.127 |
| BAFF-R on IgD+ CD38- unswitched memory B cell \|\| id:ebi-a-GCST90001707 | Inverse variance weighted | 6 | 0.029 | 0.047 | 0.542 | 1.029 | 0.939 | 1.128 |
| BAFF-R on IgD+ CD38+ B cell \|\| id:ebi-a-GCST90001708 | Inverse variance weighted | 10 | 0.043 | 0.043 | 0.313 | 1.044 | 0.960 | 1.135 |
| BAFF-R on IgD+ CD38dim B cell \|\| id:ebi-a-GCST90001709 | Inverse variance weighted | 10 | 0.040 | 0.041 | 0.332 | 1.041 | 0.960 | 1.127 |
| BAFF-R on IgD- CD24- B cell \|\| id:ebi-a-GCST90001710 | Inverse variance weighted | 8 | 0.005 | 0.046 | 0.910 | 1.005 | 0.918 | 1.101 |
| BAFF-R on IgD- CD27- B cell \|\| id:ebi-a-GCST90001711 | Inverse variance weighted | 8 | 0.005 | 0.046 | 0.912 | 1.005 | 0.918 | 1.100 |
| BAFF-R on IgD- CD38- B cell \|\| id:ebi-a-GCST90001712 | Inverse variance weighted | 7 | -0.013 | 0.048 | 0.791 | 0.987 | 0.898 | 1.085 |
| BAFF-R on IgD- CD38+ B cell \|\| id:ebi-a-GCST90001713 | Inverse variance weighted | 3 | 0.079 | 0.122 | 0.519 | 1.082 | 0.852 | 1.374 |
| BAFF-R on IgD- CD38dim B cell \|\| id:ebi-a-GCST90001714 | Wald ratio | 1 | 0.097 | 0.241 | 0.687 | 1.102 | 0.687 | 1.767 |
| BAFF-R on memory B cell \|\| id:ebi-a-GCST90001715 | Inverse variance weighted | 9 | -0.001 | 0.051 | 0.984 | 0.999 | 0.904 | 1.104 |
| BAFF-R on naive-mature B cell \|\| id:ebi-a-GCST90001716 | Inverse variance weighted | 10 | 0.040 | 0.041 | 0.326 | 1.041 | 0.960 | 1.129 |
| BAFF-R on unswitched memory B cell \|\| id:ebi-a-GCST90001717 | Inverse variance weighted | 9 | -0.001 | 0.051 | 0.987 | 0.999 | 0.905 | 1.104 |
| BAFF-R on switched memory B cell \|\| id:ebi-a-GCST90001718 | Inverse variance weighted | 10 | -0.005 | 0.043 | 0.916 | 0.995 | 0.914 | 1.084 |
| BAFF-R on IgD+ B cell \|\| id:ebi-a-GCST90001719 | Inverse variance weighted | 10 | 0.040 | 0.041 | 0.328 | 1.041 | 0.960 | 1.128 |
| BAFF-R on transitional B cell \|\| id:ebi-a-GCST90001720 | Inverse variance weighted | 8 | 0.046 | 0.045 | 0.316 | 1.047 | 0.957 | 1.144 |
| CD19 on CD20- B cell \|\| id:ebi-a-GCST90001721 | Wald ratio | 1 | 0.152 | 0.142 | 0.286 | 1.164 | 0.881 | 1.538 |
| CD19 on CD20- CD38- B cell \|\| id:ebi-a-GCST90001722 | Wald ratio | 1 | 0.341 | 0.374 | 0.362 | 1.406 | 0.676 | 2.926 |
| CD19 on CD24+ CD27+ B cell \|\| id:ebi-a-GCST90001723 | Inverse variance weighted | 3 | 0.056 | 0.084 | 0.504 | 1.058 | 0.897 | 1.246 |
| CD19 on IgD+ CD24+ B cell \|\| id:ebi-a-GCST90001724 | Inverse variance weighted | 4 | 0.092 | 0.085 | 0.280 | 1.096 | 0.928 | 1.294 |
| CD19 on IgD+ CD24- B cell \|\| id:ebi-a-GCST90001725 | Inverse variance weighted | 4 | 0.125 | 0.147 | 0.396 | 1.133 | 0.850 | 1.510 |
| CD19 on IgD+ CD38- unswitched memory B cell \|\| id:ebi-a-GCST90001728 | Inverse variance weighted | 3 | 0.232 | 0.149 | 0.120 | 1.261 | 0.941 | 1.688 |
| CD19 on IgD+ CD38+ B cell \|\| id:ebi-a-GCST90001729 | Inverse variance weighted | 4 | 0.082 | 0.078 | 0.292 | 1.085 | 0.932 | 1.264 |
| CD19 on IgD+ CD38dim B cell \|\| id:ebi-a-GCST90001730 | Inverse variance weighted | 4 | 0.095 | 0.091 | 0.300 | 1.099 | 0.919 | 1.315 |
| CD19 on IgD- CD38- B cell \|\| id:ebi-a-GCST90001733 | Wald ratio | 1 | 0.142 | 0.301 | 0.638 | 1.152 | 0.639 | 2.076 |
| CD19 on IgD- CD38dim B cell \|\| id:ebi-a-GCST90001735 | Inverse variance weighted | 4 | 0.075 | 0.079 | 0.343 | 1.078 | 0.923 | 1.259 |
| CD19 on memory B cell \|\| id:ebi-a-GCST90001736 | Inverse variance weighted | 3 | 0.065 | 0.094 | 0.492 | 1.067 | 0.887 | 1.282 |
| CD19 on naive-mature B cell \|\| id:ebi-a-GCST90001737 | Inverse variance weighted | 2 | 0.670 | 0.830 | 0.420 | 1.954 | 0.384 | 9.940 |
| CD19 on unswitched memory B cell \|\| id:ebi-a-GCST90001738 | Inverse variance weighted | 4 | 0.089 | 0.089 | 0.318 | 1.093 | 0.918 | 1.301 |
| CD19 on switched memory B cell \|\| id:ebi-a-GCST90001740 | Inverse variance weighted | 2 | 0.154 | 0.241 | 0.522 | 1.167 | 0.727 | 1.871 |
| CD19 on IgD+ B cell \|\| id:ebi-a-GCST90001741 | Inverse variance weighted | 2 | 0.335 | 0.243 | 0.168 | 1.399 | 0.868 | 2.253 |
| CD19 on transitional B cell \|\| id:ebi-a-GCST90001742 | Inverse variance weighted | 4 | 0.089 | 0.087 | 0.304 | 1.093 | 0.922 | 1.296 |
| CD20 on B cell \|\| id:ebi-a-GCST90001743 | Inverse variance weighted | 4 | -0.139 | 0.128 | 0.276 | 0.870 | 0.678 | 1.117 |
| CD20 on CD24+ CD27+ B cell \|\| id:ebi-a-GCST90001745 | Inverse variance weighted | 6 | 0.049 | 0.155 | 0.753 | 1.050 | 0.775 | 1.422 |
| CD20 on IgD+ CD24+ B cell \|\| id:ebi-a-GCST90001746 | Inverse variance weighted | 2 | -0.022 | 0.177 | 0.900 | 0.978 | 0.692 | 1.383 |
| CD20 on IgD+ CD24- B cell \|\| id:ebi-a-GCST90001747 | Inverse variance weighted | 4 | -0.119 | 0.124 | 0.337 | 0.887 | 0.695 | 1.133 |
| CD20 on IgD+ CD38- B cell \|\| id:ebi-a-GCST90001748 | Inverse variance weighted | 4 | 0.102 | 0.156 | 0.511 | 1.108 | 0.816 | 1.504 |
| CD20 on IgD+ CD38+ B cell \|\| id:ebi-a-GCST90001751 | Inverse variance weighted | 5 | -0.141 | 0.113 | 0.212 | 0.869 | 0.696 | 1.084 |
| CD20 on IgD+ CD38dim B cell \|\| id:ebi-a-GCST90001752 | Inverse variance weighted | 7 | -0.053 | 0.096 | 0.583 | 0.948 | 0.785 | 1.145 |
| CD20 on IgD- CD24- B cell \|\| id:ebi-a-GCST90001753 | Inverse variance weighted | 2 | -0.070 | 0.207 | 0.734 | 0.932 | 0.621 | 1.398 |
| CD20 on IgD- CD27- B cell \|\| id:ebi-a-GCST90001754 | Inverse variance weighted | 4 | 0.022 | 0.143 | 0.876 | 1.023 | 0.772 | 1.355 |
| CD20 on IgD- CD38- B cell \|\| id:ebi-a-GCST90001755 | Inverse variance weighted | 4 | 0.061 | 0.217 | 0.777 | 1.063 | 0.695 | 1.626 |
| CD20 on IgD- CD38dim B cell \|\| id:ebi-a-GCST90001757 | Inverse variance weighted | 7 | -0.011 | 0.103 | 0.919 | 0.990 | 0.808 | 1.212 |
| CD20 on memory B cell \|\| id:ebi-a-GCST90001758 | Inverse variance weighted | 5 | 0.099 | 0.149 | 0.507 | 1.104 | 0.824 | 1.478 |
| CD20 on naive-mature B cell \|\| id:ebi-a-GCST90001759 | Inverse variance weighted | 6 | -0.138 | 0.109 | 0.207 | 0.871 | 0.704 | 1.079 |
| CD20 on unswitched memory B cell \|\| id:ebi-a-GCST90001760 | Inverse variance weighted | 4 | -0.001 | 0.215 | 0.998 | 0.999 | 0.656 | 1.523 |
| CD20 on switched memory B cell \|\| id:ebi-a-GCST90001761 | Inverse variance weighted | 7 | 0.026 | 0.102 | 0.799 | 1.026 | 0.841 | 1.252 |
| CD20 on IgD+ B cell \|\| id:ebi-a-GCST90001762 | Inverse variance weighted | 5 | -0.153 | 0.113 | 0.176 | 0.858 | 0.687 | 1.071 |
| CD20 on transitional B cell \|\| id:ebi-a-GCST90001763 | Inverse variance weighted | 2 | 0.068 | 0.145 | 0.639 | 1.070 | 0.805 | 1.423 |
| CD24 on IgD+ CD24+ B cell \|\| id:ebi-a-GCST90001765 | Inverse variance weighted | 2 | 0.019 | 0.089 | 0.828 | 1.019 | 0.857 | 1.213 |
| CD24 on IgD+ CD38- B cell \|\| id:ebi-a-GCST90001766 | Inverse variance weighted | 2 | 0.041 | 0.143 | 0.771 | 1.042 | 0.788 | 1.378 |
| CD24 on IgD+ CD38- unswitched memory B cell \|\| id:ebi-a-GCST90001767 | Inverse variance weighted | 2 | 0.019 | 0.078 | 0.809 | 1.019 | 0.874 | 1.187 |
| CD24 on IgD+ CD38+ B cell \|\| id:ebi-a-GCST90001768 | Inverse variance weighted | 2 | -0.060 | 0.234 | 0.796 | 0.941 | 0.595 | 1.490 |
| CD24 on IgD- CD38- B cell \|\| id:ebi-a-GCST90001769 | Inverse variance weighted | 2 | 0.035 | 0.115 | 0.763 | 1.035 | 0.827 | 1.296 |
| CD24 on memory B cell \|\| id:ebi-a-GCST90001771 | Inverse variance weighted | 2 | 0.073 | 0.137 | 0.596 | 1.075 | 0.822 | 1.407 |
| CD24 on unswitched memory B cell \|\| id:ebi-a-GCST90001772 | Inverse variance weighted | 2 | 0.022 | 0.096 | 0.819 | 1.022 | 0.846 | 1.235 |
| CD24 on switched memory B cell \|\| id:ebi-a-GCST90001773 | Inverse variance weighted | 2 | 0.076 | 0.143 | 0.597 | 1.079 | 0.815 | 1.428 |
| CD24 on transitional B cell \|\| id:ebi-a-GCST90001774 | Wald ratio | 1 | 0.089 | 0.280 | 0.749 | 1.094 | 0.632 | 1.893 |
| CD25 on B cell \|\| id:ebi-a-GCST90001775 | Inverse variance weighted | 3 | 0.077 | 0.126 | 0.541 | 1.080 | 0.844 | 1.382 |
| CD25 on CD24+ CD27+ B cell \|\| id:ebi-a-GCST90001777 | Inverse variance weighted | 4 | -0.110 | 0.096 | 0.252 | 0.896 | 0.742 | 1.081 |
| CD25 on IgD+ CD24+ B cell \|\| id:ebi-a-GCST90001778 | Inverse variance weighted | 3 | -0.117 | 0.111 | 0.295 | 0.890 | 0.716 | 1.107 |
| CD25 on IgD+ CD24- B cell \|\| id:ebi-a-GCST90001779 | Inverse variance weighted | 3 | -0.024 | 0.131 | 0.854 | 0.976 | 0.755 | 1.262 |
| CD25 on IgD+ CD38- B cell \|\| id:ebi-a-GCST90001780 | Inverse variance weighted | 3 | -0.133 | 0.181 | 0.465 | 0.876 | 0.614 | 1.249 |
| CD25 on IgD+ CD38- naive B cell \|\| id:ebi-a-GCST90001781 | Inverse variance weighted | 3 | -0.042 | 0.121 | 0.730 | 0.959 | 0.757 | 1.215 |
| CD25 on IgD+ CD38- unswitched memory B cell \|\| id:ebi-a-GCST90001782 | Wald ratio | 1 | -0.145 | 0.118 | 0.217 | 0.865 | 0.687 | 1.089 |
| CD25 on IgD+ CD38+ B cell \|\| id:ebi-a-GCST90001783 | Inverse variance weighted | 3 | -0.062 | 0.137 | 0.654 | 0.940 | 0.718 | 1.231 |
| CD25 on IgD+ CD38dim B cell \|\| id:ebi-a-GCST90001784 | Inverse variance weighted | 2 | -0.036 | 0.249 | 0.885 | 0.964 | 0.591 | 1.573 |
| CD25 on IgD- CD24- B cell \|\| id:ebi-a-GCST90001785 | Inverse variance weighted | 2 | 0.071 | 0.243 | 0.769 | 1.074 | 0.666 | 1.731 |
| CD25 on IgD- CD27- B cell \|\| id:ebi-a-GCST90001786 | Inverse variance weighted | 2 | 0.000 | 0.177 | 0.999 | 1.000 | 0.707 | 1.415 |
| CD25 on IgD- CD38- B cell \|\| id:ebi-a-GCST90001787 | Inverse variance weighted | 3 | -0.038 | 0.129 | 0.769 | 0.963 | 0.747 | 1.241 |
| CD25 on IgD- CD38dim B cell \|\| id:ebi-a-GCST90001789 | Inverse variance weighted | 3 | -0.107 | 0.148 | 0.470 | 0.899 | 0.673 | 1.201 |
| CD25 on memory B cell \|\| id:ebi-a-GCST90001790 | Inverse variance weighted | 4 | -0.114 | 0.098 | 0.244 | 0.893 | 0.737 | 1.081 |
| CD25 on naive-mature B cell \|\| id:ebi-a-GCST90001791 | Inverse variance weighted | 5 | 0.082 | 0.103 | 0.425 | 1.086 | 0.887 | 1.328 |
| CD25 on unswitched memory B cell \|\| id:ebi-a-GCST90001792 | Inverse variance weighted | 4 | -0.118 | 0.101 | 0.244 | 0.889 | 0.729 | 1.084 |
| CD25 on switched memory B cell \|\| id:ebi-a-GCST90001793 | Inverse variance weighted | 3 | 0.078 | 0.115 | 0.499 | 1.081 | 0.863 | 1.354 |
| CD25 on IgD+ B cell \|\| id:ebi-a-GCST90001794 | Inverse variance weighted | 2 | 0.139 | 0.149 | 0.351 | 1.149 | 0.859 | 1.537 |
| CD25 on transitional B cell \|\| id:ebi-a-GCST90001795 | Inverse variance weighted | 2 | -0.036 | 0.177 | 0.840 | 0.965 | 0.683 | 1.364 |
| CD27 on CD24+ CD27+ B cell \|\| id:ebi-a-GCST90001798 | Inverse variance weighted | 10 | 0.013 | 0.062 | 0.841 | 1.013 | 0.896 | 1.144 |
| CD27 on T cell \|\| id:ebi-a-GCST90001799 | Wald ratio | 1 | 0.054 | 0.135 | 0.690 | 1.055 | 0.810 | 1.374 |
| CD27 on IgD+ CD24+ B cell \|\| id:ebi-a-GCST90001800 | Inverse variance weighted | 6 | 0.054 | 0.078 | 0.495 | 1.055 | 0.905 | 1.230 |
| CD27 on IgD+ CD38- unswitched memory B cell \|\| id:ebi-a-GCST90001801 | Inverse variance weighted | 5 | 0.025 | 0.065 | 0.705 | 1.025 | 0.903 | 1.164 |
| CD27 on IgD- CD38- B cell \|\| id:ebi-a-GCST90001802 | Inverse variance weighted | 7 | 0.020 | 0.073 | 0.782 | 1.021 | 0.884 | 1.179 |
| CD27 on IgD- CD38+ B cell \|\| id:ebi-a-GCST90001803 | Inverse variance weighted | 2 | -0.106 | 0.203 | 0.603 | 0.900 | 0.604 | 1.340 |
| CD27 on IgD- CD38dim B cell \|\| id:ebi-a-GCST90001804 | Inverse variance weighted | 8 | 0.038 | 0.060 | 0.522 | 1.039 | 0.924 | 1.169 |
| CD27 on memory B cell \|\| id:ebi-a-GCST90001805 | Inverse variance weighted | 7 | 0.040 | 0.073 | 0.584 | 1.041 | 0.902 | 1.202 |
| CD27 on unswitched memory B cell \|\| id:ebi-a-GCST90001806 | Inverse variance weighted | 11 | -0.005 | 0.077 | 0.949 | 0.995 | 0.857 | 1.156 |
| CD27 on Plasma Blast-Plasma Cell \|\| id:ebi-a-GCST90001807 | Wald ratio | 1 | 0.114 | 0.282 | 0.687 | 1.121 | 0.644 | 1.949 |
| CD27 on switched memory B cell \|\| id:ebi-a-GCST90001808 | Inverse variance weighted | 10 | 0.003 | 0.058 | 0.954 | 1.003 | 0.896 | 1.124 |
| CD38 on CD3- CD19- \|\| id:ebi-a-GCST90001810 | Wald ratio | 1 | -0.151 | 0.223 | 0.498 | 0.860 | 0.556 | 1.330 |
| CD38 on IgD+ CD24- B cell \|\| id:ebi-a-GCST90001811 | Inverse variance weighted | 2 | -0.045 | 0.177 | 0.801 | 0.956 | 0.676 | 1.352 |
| CD38 on IgD+ CD38+ B cell \|\| id:ebi-a-GCST90001812 | Inverse variance weighted | 3 | -0.166 | 0.123 | 0.179 | 0.847 | 0.665 | 1.079 |
| CD38 on IgD+ CD38dim B cell \|\| id:ebi-a-GCST90001813 | Inverse variance weighted | 4 | -0.045 | 0.096 | 0.637 | 0.956 | 0.792 | 1.154 |
| CD38 on IgD- CD38dim B cell \|\| id:ebi-a-GCST90001815 | Inverse variance weighted | 3 | -0.034 | 0.106 | 0.745 | 0.966 | 0.785 | 1.189 |
| CD38 on naive-mature B cell \|\| id:ebi-a-GCST90001816 | Inverse variance weighted | 3 | -0.043 | 0.117 | 0.715 | 0.958 | 0.762 | 1.205 |
| CD38 on IgD+ B cell \|\| id:ebi-a-GCST90001818 | Wald ratio | 1 | -0.341 | 0.292 | 0.244 | 0.711 | 0.401 | 1.261 |
| CD38 on transitional B cell \|\| id:ebi-a-GCST90001819 | Inverse variance weighted | 5 | -0.042 | 0.091 | 0.642 | 0.959 | 0.803 | 1.145 |
| IgD on IgD+ CD24+ B cell \|\| id:ebi-a-GCST90001820 | Inverse variance weighted | 6 | 0.017 | 0.082 | 0.838 | 1.017 | 0.866 | 1.194 |
| IgD on IgD+ CD24- B cell \|\| id:ebi-a-GCST90001821 | Inverse variance weighted | 5 | -0.018 | 0.057 | 0.747 | 0.982 | 0.878 | 1.098 |
| IgD on IgD+ CD38- B cell \|\| id:ebi-a-GCST90001822 | Wald ratio | 1 | -0.048 | 0.090 | 0.597 | 0.954 | 0.800 | 1.137 |
| IgD on IgD+ CD38- unswitched memory B cell \|\| id:ebi-a-GCST90001823 | Inverse variance weighted | 2 | -0.055 | 0.063 | 0.386 | 0.947 | 0.837 | 1.071 |
| IgD on IgD+ CD38+ B cell \|\| id:ebi-a-GCST90001824 | Inverse variance weighted | 6 | -0.063 | 0.053 | 0.230 | 0.939 | 0.847 | 1.041 |
| IgD on IgD+ CD38dim B cell \|\| id:ebi-a-GCST90001825 | Inverse variance weighted | 5 | -0.018 | 0.060 | 0.761 | 0.982 | 0.874 | 1.104 |
| IgD on unswitched memory B cell \|\| id:ebi-a-GCST90001826 | Inverse variance weighted | 6 | -0.048 | 0.071 | 0.501 | 0.953 | 0.830 | 1.095 |
| IgD on IgD+ B cell \|\| id:ebi-a-GCST90001827 | Inverse variance weighted | 2 | -0.043 | 0.073 | 0.551 | 0.958 | 0.831 | 1.104 |
| IgD on transitional B cell \|\| id:ebi-a-GCST90001828 | Inverse variance weighted | 5 | -0.056 | 0.068 | 0.411 | 0.945 | 0.827 | 1.081 |
| BAFF-R on B cell \|\| id:ebi-a-GCST90001829 | Inverse variance weighted | 10 | 0.043 | 0.041 | 0.301 | 1.044 | 0.962 | 1.132 |
| BAFF-R on CD20- B cell \|\| id:ebi-a-GCST90001830 | Wald ratio | 1 | 0.021 | 0.177 | 0.907 | 1.021 | 0.722 | 1.444 |
| CD62L on CD62L+ myeloid Dendritic Cell \|\| id:ebi-a-GCST90001831 | Inverse variance weighted | 2 | -0.166 | 0.142 | 0.244 | 0.847 | 0.642 | 1.119 |
| CD62L on CD62L+ plasmacytoid Dendritic Cell \|\| id:ebi-a-GCST90001832 | Inverse variance weighted | 3 | 0.085 | 0.147 | 0.563 | 1.089 | 0.816 | 1.453 |
| CD62L on CD62L+ Dendritic Cell \|\| id:ebi-a-GCST90001833 | Inverse variance weighted | 2 | -0.138 | 0.320 | 0.666 | 0.871 | 0.466 | 1.630 |
| CD62L on monocyte \|\| id:ebi-a-GCST90001834 | Inverse variance weighted | 2 | -0.123 | 0.128 | 0.335 | 0.884 | 0.688 | 1.136 |
| CD3 on naive CD8+ T cell \|\| id:ebi-a-GCST90001838 | Inverse variance weighted | 6 | -0.023 | 0.060 | 0.700 | 0.977 | 0.869 | 1.099 |
| CD3 on Effector Memory CD8+ T cell \|\| id:ebi-a-GCST90001839 | Inverse variance weighted | 3 | -0.094 | 0.110 | 0.391 | 0.910 | 0.734 | 1.129 |
| CD3 on Terminally Differentiated CD8+ T cell \|\| id:ebi-a-GCST90001840 | Inverse variance weighted | 2 | -0.133 | 0.164 | 0.415 | 0.875 | 0.635 | 1.206 |
| CD3 on Central Memory CD4+ T cell \|\| id:ebi-a-GCST90001841 | Inverse variance weighted | 4 | -0.034 | 0.068 | 0.612 | 0.966 | 0.846 | 1.103 |
| CD3 on Naive CD4+ T cell \|\| id:ebi-a-GCST90001842 | Inverse variance weighted | 4 | -0.029 | 0.057 | 0.609 | 0.971 | 0.868 | 1.086 |
| CD3 on Effector Memory CD4+ T cell \|\| id:ebi-a-GCST90001843 | Inverse variance weighted | 2 | -0.027 | 0.090 | 0.768 | 0.974 | 0.816 | 1.162 |
| CD3 on Terminally Differentiated CD4+ T cell \|\| id:ebi-a-GCST90001844 | Wald ratio | 1 | 0.000 | 0.174 | 0.998 | 1.000 | 0.711 | 1.406 |
| CD3 on CD45RA- CD4+ T cell \|\| id:ebi-a-GCST90001845 | Inverse variance weighted | 3 | -0.025 | 0.076 | 0.747 | 0.976 | 0.840 | 1.133 |
| CD3 on Central Memory CD8+ T cell \|\| id:ebi-a-GCST90001846 | Inverse variance weighted | 2 | -0.011 | 0.104 | 0.918 | 0.989 | 0.807 | 1.213 |
| CD3 on HLA DR+ T cell \|\| id:ebi-a-GCST90001847 | Inverse variance weighted | 2 | -0.016 | 0.109 | 0.887 | 0.985 | 0.796 | 1.218 |
| CD3 on Natural Killer T \|\| id:ebi-a-GCST90001848 | Inverse variance weighted | 2 | -0.119 | 0.228 | 0.600 | 0.887 | 0.568 | 1.387 |
| CD3 on HLA DR+ CD4+ T cell \|\| id:ebi-a-GCST90001849 | Inverse variance weighted | 2 | 0.090 | 0.267 | 0.736 | 1.094 | 0.648 | 1.846 |
| CD3 on HLA DR+ CD8+ T cell \|\| id:ebi-a-GCST90001850 | Wald ratio | 1 | -0.006 | 0.170 | 0.973 | 0.994 | 0.713 | 1.387 |
| CD3 on T cell \|\| id:ebi-a-GCST90001851 | Inverse variance weighted | 3 | -0.008 | 0.088 | 0.932 | 0.992 | 0.835 | 1.180 |
| CD3 on CD39+ resting CD4 regulatory T cell \|\| id:ebi-a-GCST90001852 | Wald ratio | 1 | -0.005 | 0.146 | 0.975 | 0.995 | 0.748 | 1.325 |
| CD3 on activated CD4 regulatory T cell \|\| id:ebi-a-GCST90001853 | Inverse variance weighted | 4 | -0.010 | 0.065 | 0.876 | 0.990 | 0.872 | 1.124 |
| CD3 on CD39+ activated CD4 regulatory T cell \|\| id:ebi-a-GCST90001854 | Inverse variance weighted | 5 | 0.020 | 0.065 | 0.755 | 1.020 | 0.899 | 1.158 |
| CD3 on secreting CD4 regulatory T cell \|\| id:ebi-a-GCST90001855 | Inverse variance weighted | 3 | -0.047 | 0.069 | 0.494 | 0.954 | 0.833 | 1.092 |
| CD3 on CD39+ secreting CD4 regulatory T cell \|\| id:ebi-a-GCST90001856 | Inverse variance weighted | 3 | -0.052 | 0.078 | 0.502 | 0.949 | 0.815 | 1.105 |
| CD3 on activated & secreting CD4 regulatory T cell \|\| id:ebi-a-GCST90001857 | Inverse variance weighted | 4 | -0.011 | 0.064 | 0.859 | 0.989 | 0.873 | 1.120 |
| CD3 on CD45RA+ CD4+ T cell \|\| id:ebi-a-GCST90001858 | Inverse variance weighted | 5 | -0.034 | 0.058 | 0.558 | 0.967 | 0.863 | 1.083 |
| CD3 on CD8+ T cell \|\| id:ebi-a-GCST90001859 | Wald ratio | 1 | 0.000 | 0.116 | 0.998 | 1.000 | 0.797 | 1.254 |
| CD3 on CD39+ CD4+ T cell \|\| id:ebi-a-GCST90001860 | Inverse variance weighted | 4 | 0.022 | 0.108 | 0.842 | 1.022 | 0.827 | 1.263 |
| CD3 on CD28+ CD4+ T cell \|\| id:ebi-a-GCST90001861 | Inverse variance weighted | 2 | 0.003 | 0.085 | 0.968 | 1.003 | 0.850 | 1.185 |
| CD3 on CD28+ CD45RA- CD8+ T cell \|\| id:ebi-a-GCST90001863 | Inverse variance weighted | 4 | 0.020 | 0.148 | 0.895 | 1.020 | 0.763 | 1.364 |
| CD3 on CD28+ CD45RA+ CD8+ T cell \|\| id:ebi-a-GCST90001864 | Inverse variance weighted | 3 | -0.006 | 0.101 | 0.956 | 0.994 | 0.815 | 1.213 |
| CD3 on CD28- CD8+ T cell \|\| id:ebi-a-GCST90001865 | Inverse variance weighted | 2 | -0.090 | 0.150 | 0.548 | 0.914 | 0.682 | 1.226 |
| CD3 on CD39+ CD8+ T cell \|\| id:ebi-a-GCST90001866 | Wald ratio | 1 | 0.000 | 0.136 | 0.998 | 1.000 | 0.766 | 1.305 |
| CD3 on CD4+ T cell \|\| id:ebi-a-GCST90001867 | Inverse variance weighted | 2 | -0.030 | 0.240 | 0.902 | 0.971 | 0.606 | 1.554 |
| CD3 on CD4 regulatory T cell \|\| id:ebi-a-GCST90001868 | Inverse variance weighted | 3 | -0.046 | 0.068 | 0.497 | 0.955 | 0.835 | 1.091 |
| CD3 on resting CD4 regulatory T cell \|\| id:ebi-a-GCST90001869 | Inverse variance weighted | 5 | 0.046 | 0.073 | 0.526 | 1.047 | 0.908 | 1.207 |
| CD34 on Hematopoietic Stem Cell \|\| id:ebi-a-GCST90001870 | Inverse variance weighted | 4 | -0.084 | 0.116 | 0.470 | 0.920 | 0.733 | 1.154 |
| HVEM on T cell \|\| id:ebi-a-GCST90001871 | Wald ratio | 1 | 0.050 | 0.147 | 0.736 | 1.051 | 0.788 | 1.402 |
| HVEM on naive CD8+ T cell \|\| id:ebi-a-GCST90001872 | Inverse variance weighted | 2 | -0.008 | 0.142 | 0.958 | 0.992 | 0.751 | 1.311 |
| HVEM on Effector Memory CD8+ T cell \|\| id:ebi-a-GCST90001873 | Wald ratio | 1 | 0.050 | 0.147 | 0.736 | 1.051 | 0.787 | 1.403 |
| HVEM on CD4+ T cell \|\| id:ebi-a-GCST90001875 | Wald ratio | 1 | 0.048 | 0.144 | 0.736 | 1.050 | 0.792 | 1.391 |
| HVEM on Central Memory CD4+ T cell \|\| id:ebi-a-GCST90001876 | Wald ratio | 1 | 0.050 | 0.147 | 0.736 | 1.051 | 0.787 | 1.403 |
| HVEM on naive CD4+ T cell \|\| id:ebi-a-GCST90001877 | Wald ratio | 1 | 0.101 | 0.169 | 0.550 | 1.106 | 0.794 | 1.541 |
| HVEM on Effector Memory CD4+ T cell \|\| id:ebi-a-GCST90001878 | Wald ratio | 1 | 0.055 | 0.146 | 0.706 | 1.057 | 0.794 | 1.407 |
| HVEM on Terminally Differentiated CD4+ T cell \|\| id:ebi-a-GCST90001879 | Wald ratio | 1 | 0.095 | 0.176 | 0.589 | 1.100 | 0.779 | 1.554 |
| HVEM on CD45RA- CD4+ T cell \|\| id:ebi-a-GCST90001880 | Wald ratio | 1 | 0.047 | 0.140 | 0.736 | 1.048 | 0.797 | 1.380 |
| HVEM on CD8+ T cell \|\| id:ebi-a-GCST90001881 | Wald ratio | 1 | 0.088 | 0.163 | 0.589 | 1.092 | 0.794 | 1.501 |
| HVEM on Central Memory CD8+ T cell \|\| id:ebi-a-GCST90001882 | Wald ratio | 1 | 0.046 | 0.136 | 0.736 | 1.047 | 0.802 | 1.367 |
| CD16-CD56 on Natural Killer T \|\| id:ebi-a-GCST90001883 | Inverse variance weighted | 5 | 0.134 | 0.120 | 0.263 | 1.144 | 0.904 | 1.447 |
| CD16-CD56 on Natural Killer \|\| id:ebi-a-GCST90001884 | Inverse variance weighted | 8 | 0.083 | 0.087 | 0.342 | 1.086 | 0.916 | 1.289 |
| CD16-CD56 on HLA DR+ Natural Killer \|\| id:ebi-a-GCST90001885 | Inverse variance weighted | 2 | 0.655 | 0.254 | 0.010 | 1.925 | 1.171 | 3.165 |
| CD28 on CD39+ activated CD4 regulatory T cell \|\| id:ebi-a-GCST90001886 | Inverse variance weighted | 3 | -0.113 | 0.116 | 0.331 | 0.893 | 0.712 | 1.122 |
| CD28 on secreting CD4 regulatory T cell \|\| id:ebi-a-GCST90001887 | Inverse variance weighted | 3 | 0.101 | 0.130 | 0.437 | 1.106 | 0.858 | 1.425 |
| CD28 on CD39+ secreting CD4 regulatory T cell \|\| id:ebi-a-GCST90001888 | Inverse variance weighted | 2 | -0.032 | 0.101 | 0.750 | 0.968 | 0.795 | 1.180 |
| CD28 on activated & secreting CD4 regulatory T cell \|\| id:ebi-a-GCST90001889 | Wald ratio | 1 | 0.177 | 0.156 | 0.258 | 1.193 | 0.879 | 1.620 |
| CD28 on CD45RA+ CD4+ T cell \|\| id:ebi-a-GCST90001890 | Inverse variance weighted | 2 | 0.225 | 0.116 | 0.053 | 1.252 | 0.997 | 1.573 |
| CD28 on CD45RA- CD4 not regulatory T cell \|\| id:ebi-a-GCST90001891 | Inverse variance weighted | 3 | 0.153 | 0.117 | 0.190 | 1.165 | 0.927 | 1.465 |
| CD28 on CD39+ CD4+ T cell \|\| id:ebi-a-GCST90001892 | Inverse variance weighted | 5 | -0.009 | 0.067 | 0.888 | 0.991 | 0.869 | 1.129 |
| CD28 on CD28+ CD45RA- CD8+ T cell \|\| id:ebi-a-GCST90001893 | Wald ratio | 1 | 0.132 | 0.132 | 0.317 | 1.142 | 0.881 | 1.480 |
| CD28 on CD28+ CD4+ T cell \|\| id:ebi-a-GCST90001894 | Inverse variance weighted | 2 | 0.134 | 0.153 | 0.379 | 1.144 | 0.848 | 1.543 |
| CD28 on CD28+ CD45RA+ CD8+ T cell \|\| id:ebi-a-GCST90001896 | Inverse variance weighted | 3 | 0.148 | 0.133 | 0.268 | 1.159 | 0.893 | 1.505 |
| CD28 on CD39+ CD8+ T cell \|\| id:ebi-a-GCST90001897 | Inverse variance weighted | 2 | 0.126 | 0.053 | 0.018 | 1.134 | 1.022 | 1.258 |
| CD28 on CD4+ T cell \|\| id:ebi-a-GCST90001898 | Inverse variance weighted | 2 | 0.141 | 0.163 | 0.388 | 1.151 | 0.836 | 1.585 |
| CD28 on CD4 regulatory T cell \|\| id:ebi-a-GCST90001899 | Wald ratio | 1 | 0.176 | 0.179 | 0.324 | 1.192 | 0.840 | 1.692 |
| CD28 on resting CD4 regulatory T cell \|\| id:ebi-a-GCST90001900 | Wald ratio | 1 | 0.143 | 0.122 | 0.245 | 1.153 | 0.907 | 1.466 |
| CD28 on CD39+ resting CD4 regulatory T cell \|\| id:ebi-a-GCST90001901 | Inverse variance weighted | 2 | -0.041 | 0.180 | 0.821 | 0.960 | 0.674 | 1.367 |
| CD28 on activated CD4 regulatory T cell \|\| id:ebi-a-GCST90001902 | Wald ratio | 1 | 0.201 | 0.177 | 0.258 | 1.222 | 0.864 | 1.730 |
| CD86 on myeloid Dendritic Cell \|\| id:ebi-a-GCST90001903 | Wald ratio | 1 | 0.284 | 0.129 | 0.028 | 1.328 | 1.032 | 1.710 |
| CD86 on CD62L+ myeloid Dendritic Cell \|\| id:ebi-a-GCST90001904 | Wald ratio | 1 | 0.088 | 0.112 | 0.434 | 1.092 | 0.877 | 1.359 |
| CD86 on monocyte \|\| id:ebi-a-GCST90001905 | Wald ratio | 1 | -0.413 | 0.284 | 0.145 | 0.662 | 0.379 | 1.154 |
| CD86 on granulocyte \|\| id:ebi-a-GCST90001906 | Inverse variance weighted | 2 | 0.223 | 0.324 | 0.491 | 1.250 | 0.663 | 2.357 |
| CCR7 on naive CD4+ T cell \|\| id:ebi-a-GCST90001907 | Inverse variance weighted | 4 | -0.002 | 0.099 | 0.985 | 0.998 | 0.822 | 1.212 |
| CCR7 on naive CD8+ T cell \|\| id:ebi-a-GCST90001908 | Inverse variance weighted | 2 | 0.140 | 0.177 | 0.430 | 1.150 | 0.813 | 1.627 |
| CD45 on CD14+ monocyte \|\| id:ebi-a-GCST90001909 | Wald ratio | 1 | -0.113 | 0.485 | 0.816 | 0.893 | 0.345 | 2.310 |
| CD45 on B cell \|\| id:ebi-a-GCST90001910 | Inverse variance weighted | 2 | -0.140 | 0.233 | 0.549 | 0.870 | 0.551 | 1.373 |
| CD45 on Natural Killer \|\| id:ebi-a-GCST90001911 | Inverse variance weighted | 2 | 0.384 | 0.187 | 0.040 | 1.468 | 1.018 | 2.119 |
| CD45 on granulocyte \|\| id:ebi-a-GCST90001913 | Wald ratio | 1 | 0.160 | 0.269 | 0.552 | 1.174 | 0.692 | 1.990 |
| CD45 on CD4+ T cell \|\| id:ebi-a-GCST90001916 | Wald ratio | 1 | 0.043 | 0.250 | 0.863 | 1.044 | 0.639 | 1.706 |
| CD45 on CD8+ T cell \|\| id:ebi-a-GCST90001917 | Inverse variance weighted | 2 | 0.069 | 0.097 | 0.473 | 1.072 | 0.887 | 1.295 |
| CD45 on HLA DR+ T cell \|\| id:ebi-a-GCST90001918 | Wald ratio | 1 | -0.055 | 0.170 | 0.745 | 0.946 | 0.678 | 1.320 |
| CD45 on Natural Killer T \|\| id:ebi-a-GCST90001919 | Wald ratio | 1 | 0.132 | 0.276 | 0.634 | 1.141 | 0.664 | 1.959 |
| CD45 on HLA DR+ CD4+ T cell \|\| id:ebi-a-GCST90001920 | Inverse variance weighted | 2 | -0.285 | 0.262 | 0.278 | 0.752 | 0.450 | 1.258 |
| CD127 on CD45RA- CD4 not regulatory T cell \|\| id:ebi-a-GCST90001923 | Wald ratio | 1 | 0.124 | 0.280 | 0.659 | 1.132 | 0.653 | 1.961 |
| CD127 on CD28+ CD4+ T cell \|\| id:ebi-a-GCST90001924 | Inverse variance weighted | 3 | 0.190 | 0.146 | 0.191 | 1.210 | 0.909 | 1.609 |
| CD127 on granulocyte \|\| id:ebi-a-GCST90001926 | Inverse variance weighted | 8 | -0.009 | 0.069 | 0.894 | 0.991 | 0.866 | 1.134 |
| CD127 on CD8+ T cell \|\| id:ebi-a-GCST90001927 | Wald ratio | 1 | 0.522 | 0.233 | 0.025 | 1.686 | 1.068 | 2.661 |
| CD127 on CD28+ CD45RA- CD8+ T cell \|\| id:ebi-a-GCST90001928 | Wald ratio | 1 | -0.037 | 0.103 | 0.720 | 0.964 | 0.788 | 1.179 |
| CD127 on CD28+ CD45RA+ CD8+ T cell \|\| id:ebi-a-GCST90001929 | Inverse variance weighted | 2 | 0.110 | 0.169 | 0.514 | 1.116 | 0.802 | 1.554 |
| CD127 on CD45RA+ CD4+ T cell \|\| id:ebi-a-GCST90001932 | Inverse variance weighted | 4 | -0.021 | 0.107 | 0.846 | 0.979 | 0.794 | 1.208 |
| CD25 on CD45RA- CD4 not regulatory T cell \|\| id:ebi-a-GCST90001933 | Inverse variance weighted | 2 | 0.065 | 0.163 | 0.689 | 1.067 | 0.776 | 1.468 |
| CD25 on CD45RA+ CD4 not regulatory T cell \|\| id:ebi-a-GCST90001934 | Wald ratio | 1 | 0.146 | 0.191 | 0.445 | 1.157 | 0.796 | 1.683 |
| CD25 on CD39+ CD4 regulatory T cell \|\| id:ebi-a-GCST90001935 | Wald ratio | 1 | -0.143 | 0.240 | 0.550 | 0.866 | 0.541 | 1.387 |
| CD25 on resting CD4 regulatory T cell \|\| id:ebi-a-GCST90001937 | Wald ratio | 1 | 0.917 | 1.225 | 0.454 | 2.502 | 0.227 | 27.615 |
| CD25 on CD39+ resting CD4 regulatory T cell \|\| id:ebi-a-GCST90001938 | Wald ratio | 1 | -0.185 | 0.363 | 0.611 | 0.831 | 0.408 | 1.695 |
| CD25 on CD39+ activated CD4 regulatory T cell \|\| id:ebi-a-GCST90001940 | Wald ratio | 1 | -0.152 | 0.255 | 0.550 | 0.859 | 0.521 | 1.415 |
| CD25 on secreting CD4 regulatory T cell \|\| id:ebi-a-GCST90001941 | Inverse variance weighted | 2 | -0.383 | 0.143 | 0.007 | 0.682 | 0.516 | 0.903 |
| CD25 on CD39+ secreting CD4 regulatory T cell \|\| id:ebi-a-GCST90001942 | Inverse variance weighted | 2 | -0.399 | 0.149 | 0.008 | 0.671 | 0.501 | 0.900 |
| CD25 on activated & secreting CD4 regulatory T cell \|\| id:ebi-a-GCST90001943 | Wald ratio | 1 | -0.416 | 0.192 | 0.030 | 0.660 | 0.453 | 0.962 |
| CD123 on plasmacytoid Dendritic Cell \|\| id:ebi-a-GCST90001944 | Inverse variance weighted | 2 | -0.054 | 0.136 | 0.691 | 0.947 | 0.725 | 1.237 |
| CD123 on CD62L+ plasmacytoid Dendritic Cell \|\| id:ebi-a-GCST90001945 | Inverse variance weighted | 2 | -0.055 | 0.137 | 0.690 | 0.947 | 0.723 | 1.239 |
| CD33 on CD14+ monocyte \|\| id:ebi-a-GCST90001946 | Inverse variance weighted | 5 | 0.059 | 0.035 | 0.096 | 1.060 | 0.990 | 1.136 |
| CD33 on CD33+ HLA DR+ CD14dim \|\| id:ebi-a-GCST90001947 | Inverse variance weighted | 4 | 0.046 | 0.041 | 0.264 | 1.047 | 0.966 | 1.135 |
| CD33 on CD33dim HLA DR+ CD11b+ \|\| id:ebi-a-GCST90001948 | Inverse variance weighted | 6 | 0.055 | 0.034 | 0.113 | 1.056 | 0.987 | 1.130 |
| CD33 on CD33dim HLA DR+ CD11b- \|\| id:ebi-a-GCST90001949 | Inverse variance weighted | 7 | 0.042 | 0.034 | 0.223 | 1.043 | 0.975 | 1.115 |
| CD33 on Granulocytic Myeloid-Derived Suppressor Cells \|\| id:ebi-a-GCST90001950 | Inverse variance weighted | 3 | 0.088 | 0.049 | 0.072 | 1.092 | 0.992 | 1.201 |
| CD33 on CD66b++ myeloid cell \|\| id:ebi-a-GCST90001951 | Inverse variance weighted | 4 | 0.079 | 0.041 | 0.056 | 1.082 | 0.998 | 1.174 |
| CD33 on Monocytic Myeloid-Derived Suppressor Cells \|\| id:ebi-a-GCST90001952 | Inverse variance weighted | 4 | 0.049 | 0.043 | 0.249 | 1.051 | 0.966 | 1.143 |
| CD33 on CD33dim HLA DR- \|\| id:ebi-a-GCST90001953 | Inverse variance weighted | 6 | 0.059 | 0.035 | 0.097 | 1.060 | 0.989 | 1.136 |
| CD33 on basophil \|\| id:ebi-a-GCST90001954 | Inverse variance weighted | 7 | 0.056 | 0.038 | 0.143 | 1.058 | 0.981 | 1.140 |
| CD33 on Immature Myeloid-Derived Suppressor Cells \|\| id:ebi-a-GCST90001955 | Inverse variance weighted | 6 | 0.064 | 0.041 | 0.116 | 1.066 | 0.984 | 1.154 |
| CD33 on CD33+ HLA DR+ \|\| id:ebi-a-GCST90001956 | Inverse variance weighted | 4 | 0.045 | 0.041 | 0.266 | 1.047 | 0.966 | 1.134 |
| CD33 on CD33+ HLA DR+ CD14- \|\| id:ebi-a-GCST90001957 | Inverse variance weighted | 4 | 0.045 | 0.041 | 0.271 | 1.046 | 0.966 | 1.133 |
| CD4 on monocyte \|\| id:ebi-a-GCST90001958 | Wald ratio | 1 | -0.028 | 0.188 | 0.882 | 0.973 | 0.673 | 1.405 |
| CD4 on HLA DR+ CD4+ T cell \|\| id:ebi-a-GCST90001959 | Inverse variance weighted | 2 | 0.106 | 0.201 | 0.596 | 1.112 | 0.750 | 1.648 |
| CD25 on CD4+ T cell \|\| id:ebi-a-GCST90001960 | Wald ratio | 1 | 0.110 | 0.276 | 0.691 | 1.116 | 0.650 | 1.915 |
| CD25 on CD39+ CD4+ T cell \|\| id:ebi-a-GCST90001961 | Inverse variance weighted | 6 | 0.008 | 0.056 | 0.888 | 1.008 | 0.904 | 1.124 |
| FSC-A on myeloid Dendritic Cell \|\| id:ebi-a-GCST90001963 | Inverse variance weighted | 3 | -0.090 | 0.141 | 0.523 | 0.914 | 0.693 | 1.205 |
| FSC-A on plasmacytoid Dendritic Cell \|\| id:ebi-a-GCST90001964 | Inverse variance weighted | 4 | 0.179 | 0.165 | 0.278 | 1.196 | 0.866 | 1.651 |
| FSC-A on monocyte \|\| id:ebi-a-GCST90001965 | Inverse variance weighted | 2 | 0.035 | 0.267 | 0.896 | 1.036 | 0.614 | 1.747 |
| FSC-A on granulocyte \|\| id:ebi-a-GCST90001966 | Inverse variance weighted | 2 | -0.204 | 0.310 | 0.510 | 0.815 | 0.445 | 1.496 |
| FSC-A on CD14+ monocyte \|\| id:ebi-a-GCST90001967 | Wald ratio | 1 | 0.110 | 0.625 | 0.861 | 1.116 | 0.328 | 3.795 |
| FSC-A on Natural Killer \|\| id:ebi-a-GCST90001969 | Inverse variance weighted | 3 | -0.048 | 0.153 | 0.755 | 0.953 | 0.706 | 1.288 |
| FSC-A on HLA DR+ Natural Killer \|\| id:ebi-a-GCST90001970 | Inverse variance weighted | 4 | -0.174 | 0.107 | 0.103 | 0.840 | 0.681 | 1.036 |
| FSC-A on CD4+ T cell \|\| id:ebi-a-GCST90001973 | Inverse variance weighted | 3 | 0.123 | 0.305 | 0.686 | 1.131 | 0.622 | 2.056 |
| FSC-A on CD8+ T cell \|\| id:ebi-a-GCST90001974 | Wald ratio | 1 | -0.183 | 0.540 | 0.735 | 0.833 | 0.289 | 2.401 |
| FSC-A on Natural Killer T \|\| id:ebi-a-GCST90001976 | Inverse variance weighted | 2 | -0.257 | 0.264 | 0.330 | 0.773 | 0.461 | 1.297 |
| CD16 on CD14- CD16+ monocyte \|\| id:ebi-a-GCST90001979 | Inverse variance weighted | 8 | -0.084 | 0.049 | 0.089 | 0.920 | 0.835 | 1.013 |
| CD40 on CD14+ CD16- monocyte \|\| id:ebi-a-GCST90001980 | Inverse variance weighted | 4 | -0.034 | 0.056 | 0.546 | 0.967 | 0.866 | 1.079 |
| CD40 on CD14+ CD16+ monocyte \|\| id:ebi-a-GCST90001981 | Inverse variance weighted | 5 | -0.014 | 0.051 | 0.785 | 0.986 | 0.893 | 1.089 |
| CCR2 on CD14- CD16+ monocyte \|\| id:ebi-a-GCST90001982 | Wald ratio | 1 | 0.616 | 0.321 | 0.055 | 1.852 | 0.987 | 3.476 |
| CD14 on CD14+ CD16+ monocyte \|\| id:ebi-a-GCST90001983 | Inverse variance weighted | 2 | -0.055 | 0.178 | 0.757 | 0.947 | 0.668 | 1.341 |
| HLA DR on CD14- CD16+ monocyte \|\| id:ebi-a-GCST90001984 | Inverse variance weighted | 8 | -0.075 | 0.058 | 0.193 | 0.927 | 0.828 | 1.039 |
| CD40 on monocytes \|\| id:ebi-a-GCST90001985 | Inverse variance weighted | 5 | -0.006 | 0.050 | 0.910 | 0.994 | 0.901 | 1.097 |
| CD14 on CD14+ CD16- monocyte \|\| id:ebi-a-GCST90001986 | Wald ratio | 1 | -0.288 | 0.183 | 0.115 | 0.750 | 0.524 | 1.073 |
| CD64 on CD14+ CD16- monocyte \|\| id:ebi-a-GCST90001987 | Inverse variance weighted | 8 | -0.008 | 0.053 | 0.873 | 0.992 | 0.894 | 1.100 |
| HLA DR on CD14+ CD16- monocyte \|\| id:ebi-a-GCST90001988 | Inverse variance weighted | 5 | -0.069 | 0.061 | 0.255 | 0.933 | 0.829 | 1.051 |
| CD40 on CD14- CD16+ monocyte \|\| id:ebi-a-GCST90001989 | Inverse variance weighted | 9 | -0.003 | 0.047 | 0.957 | 0.997 | 0.910 | 1.094 |
| CD64 on CD14- CD16+ monocyte \|\| id:ebi-a-GCST90001990 | Inverse variance weighted | 2 | 0.112 | 0.485 | 0.818 | 1.118 | 0.432 | 2.894 |
| HLA DR on CD14+ monocyte \|\| id:ebi-a-GCST90001991 | Inverse variance weighted | 5 | -0.071 | 0.062 | 0.258 | 0.932 | 0.825 | 1.053 |
| CCR2 on CD14+ CD16+ monocyte \|\| id:ebi-a-GCST90001992 | Inverse variance weighted | 2 | 0.704 | 0.268 | 0.009 | 2.022 | 1.196 | 3.417 |
| PDL-1 on CD14+ CD16- monocyte \|\| id:ebi-a-GCST90001993 | Wald ratio | 1 | -0.014 | 0.302 | 0.964 | 0.987 | 0.546 | 1.784 |
| CX3CR1 on CD14- CD16- \|\| id:ebi-a-GCST90001994 | Inverse variance weighted | 3 | 0.355 | 0.138 | 0.010 | 1.427 | 1.088 | 1.870 |
| CX3CR1 on monocyte \|\| id:ebi-a-GCST90001995 | Inverse variance weighted | 3 | 0.099 | 0.082 | 0.231 | 1.104 | 0.939 | 1.297 |
| CX3CR1 on CD14+ CD16+ monocyte \|\| id:ebi-a-GCST90001996 | Inverse variance weighted | 3 | 0.081 | 0.082 | 0.323 | 1.084 | 0.924 | 1.273 |
| CX3CR1 on CD14+ CD16- monocyte \|\| id:ebi-a-GCST90001997 | Inverse variance weighted | 3 | 0.089 | 0.075 | 0.232 | 1.094 | 0.944 | 1.266 |
| PDL-1 on CD14+ CD16+ monocyte \|\| id:ebi-a-GCST90001998 | Wald ratio | 1 | -0.115 | 0.290 | 0.690 | 0.891 | 0.505 | 1.572 |
| PDL-1 on CD14- CD16+ monocyte \|\| id:ebi-a-GCST90001999 | Inverse variance weighted | 2 | -0.245 | 0.113 | 0.030 | 0.783 | 0.628 | 0.976 |
| PDL-1 on CD14- CD16- \|\| id:ebi-a-GCST90002000 | Wald ratio | 1 | 0.361 | 0.349 | 0.301 | 1.435 | 0.724 | 2.845 |
| CD64 on CD14- CD16- \|\| id:ebi-a-GCST90002001 | Inverse variance weighted | 3 | -0.266 | 0.138 | 0.053 | 0.766 | 0.585 | 1.004 |
| PDL-1 on monocyte \|\| id:ebi-a-GCST90002002 | Inverse variance weighted | 2 | -0.260 | 0.129 | 0.044 | 0.771 | 0.599 | 0.993 |
| CCR2 on CD14- CD16- \|\| id:ebi-a-GCST90002003 | Inverse variance weighted | 3 | 0.391 | 0.176 | 0.027 | 1.479 | 1.047 | 2.089 |
| CCR2 on CD14+ CD16- monocyte \|\| id:ebi-a-GCST90002004 | Inverse variance weighted | 3 | 0.070 | 0.119 | 0.558 | 1.073 | 0.849 | 1.355 |
| CD16 on CD14+ CD16+ monocyte \|\| id:ebi-a-GCST90002005 | Inverse variance weighted | 5 | -0.127 | 0.071 | 0.076 | 0.881 | 0.766 | 1.013 |
| CD64 on monocyte \|\| id:ebi-a-GCST90002006 | Inverse variance weighted | 8 | -0.010 | 0.057 | 0.864 | 0.990 | 0.886 | 1.107 |
| HLA DR on CD14+ CD16+ monocyte \|\| id:ebi-a-GCST90002007 | Inverse variance weighted | 5 | -0.151 | 0.068 | 0.025 | 0.860 | 0.753 | 0.982 |
| CCR2 on monocyte \|\| id:ebi-a-GCST90002008 | Inverse variance weighted | 2 | 0.076 | 0.161 | 0.636 | 1.079 | 0.788 | 1.478 |
| HLA DR on CD14- CD16- \|\| id:ebi-a-GCST90002009 | Inverse variance weighted | 5 | -0.055 | 0.060 | 0.358 | 0.946 | 0.841 | 1.065 |
| HLA DR on monocyte \|\| id:ebi-a-GCST90002010 | Inverse variance weighted | 5 | -0.071 | 0.065 | 0.275 | 0.931 | 0.819 | 1.058 |
| CD64 on CD14+ CD16+ monocyte \|\| id:ebi-a-GCST90002011 | Inverse variance weighted | 2 | 0.483 | 0.279 | 0.084 | 1.620 | 0.937 | 2.801 |
| CX3CR1 on CD14- CD16+ monocyte \|\| id:ebi-a-GCST90002012 | Inverse variance weighted | 2 | -0.015 | 0.339 | 0.965 | 0.985 | 0.507 | 1.914 |
| CCR2 on myeloid Dendritic Cell \|\| id:ebi-a-GCST90002013 | Inverse variance weighted | 3 | -0.078 | 0.126 | 0.535 | 0.925 | 0.723 | 1.183 |
| CCR2 on CD62L+ myeloid Dendritic Cell \|\| id:ebi-a-GCST90002014 | Inverse variance weighted | 3 | 0.138 | 0.090 | 0.125 | 1.148 | 0.962 | 1.370 |
| CCR2 on plasmacytoid Dendritic Cell \|\| id:ebi-a-GCST90002015 | Inverse variance weighted | 2 | 0.141 | 0.105 | 0.176 | 1.152 | 0.939 | 1.414 |
| CCR2 on CD62L+ plasmacytoid Dendritic Cell \|\| id:ebi-a-GCST90002016 | Inverse variance weighted | 2 | 0.142 | 0.105 | 0.176 | 1.152 | 0.938 | 1.415 |
| CCR2 on monocyte \|\| id:ebi-a-GCST90002017 | Inverse variance weighted | 2 | 0.013 | 0.103 | 0.901 | 1.013 | 0.828 | 1.240 |
| CCR2 on granulocyte \|\| id:ebi-a-GCST90002018 | Inverse variance weighted | 2 | -0.339 | 0.316 | 0.283 | 0.712 | 0.383 | 1.323 |
| CD14 on Monocytic Myeloid-Derived Suppressor Cells \|\| id:ebi-a-GCST90002019 | Wald ratio | 1 | 0.038 | 0.148 | 0.796 | 1.039 | 0.778 | 1.388 |
| CD14 on CD33dim HLA DR+ CD11b+ \|\| id:ebi-a-GCST90002021 | Wald ratio | 1 | 0.049 | 0.124 | 0.690 | 1.051 | 0.824 | 1.340 |
| CD4 on CD4+ T cell \|\| id:ebi-a-GCST90002022 | Wald ratio | 1 | 0.078 | 0.169 | 0.644 | 1.081 | 0.776 | 1.506 |
| CD4 on Central Memory CD4+ T cell \|\| id:ebi-a-GCST90002023 | Inverse variance weighted | 2 | 0.018 | 0.127 | 0.888 | 1.018 | 0.793 | 1.306 |
| CD4 on naive CD4+ T cell \|\| id:ebi-a-GCST90002024 | Inverse variance weighted | 5 | 0.175 | 0.121 | 0.148 | 1.191 | 0.940 | 1.508 |
| CD4 on Effector Memory CD4+ T cell \|\| id:ebi-a-GCST90002025 | Inverse variance weighted | 3 | 0.079 | 0.101 | 0.434 | 1.083 | 0.887 | 1.321 |
| CD4 on CD45RA+ CD4+ T cell \|\| id:ebi-a-GCST90002027 | Inverse variance weighted | 2 | 0.016 | 0.118 | 0.892 | 1.016 | 0.807 | 1.279 |
| CD19 on B cell \|\| id:ebi-a-GCST90002028 | Wald ratio | 1 | 0.015 | 0.269 | 0.956 | 1.015 | 0.599 | 1.721 |
| CD39 on CD39+ CD8+ T cell \|\| id:ebi-a-GCST90002029 | Inverse variance weighted | 2 | -0.017 | 0.163 | 0.915 | 0.983 | 0.714 | 1.353 |
| CD39 on CD39+ activated CD4 regulatory T cell \|\| id:ebi-a-GCST90002030 | Inverse variance weighted | 6 | 0.003 | 0.031 | 0.932 | 1.003 | 0.943 | 1.066 |
| CD39 on CD39+ secreting CD4 regulatory T cell \|\| id:ebi-a-GCST90002031 | Inverse variance weighted | 6 | 0.004 | 0.036 | 0.922 | 1.004 | 0.935 | 1.078 |
| CD39 on CD39+ CD4+ T cell \|\| id:ebi-a-GCST90002032 | Inverse variance weighted | 5 | 0.011 | 0.036 | 0.761 | 1.011 | 0.942 | 1.085 |
| CD39 on granulocyte \|\| id:ebi-a-GCST90002033 | Inverse variance weighted | 2 | 0.289 | 0.246 | 0.240 | 1.335 | 0.825 | 2.159 |
| CD39 on monocyte \|\| id:ebi-a-GCST90002034 | Inverse variance weighted | 2 | -0.035 | 0.253 | 0.889 | 0.965 | 0.588 | 1.586 |
| CD80 on myeloid Dendritic Cell \|\| id:ebi-a-GCST90002035 | Inverse variance weighted | 4 | 0.019 | 0.205 | 0.925 | 1.020 | 0.682 | 1.524 |
| CD80 on CD62L+ myeloid Dendritic Cell \|\| id:ebi-a-GCST90002036 | Inverse variance weighted | 3 | -0.154 | 0.148 | 0.296 | 0.857 | 0.642 | 1.144 |
| CD80 on plasmacytoid Dendritic Cell \|\| id:ebi-a-GCST90002037 | Inverse variance weighted | 2 | -0.058 | 0.144 | 0.688 | 0.944 | 0.711 | 1.253 |
| CD80 on CD62L+ plasmacytoid Dendritic Cell \|\| id:ebi-a-GCST90002038 | Inverse variance weighted | 2 | -0.059 | 0.146 | 0.687 | 0.943 | 0.709 | 1.255 |
| CD80 on monocyte \|\| id:ebi-a-GCST90002039 | Inverse variance weighted | 6 | -0.153 | 0.068 | 0.026 | 0.859 | 0.751 | 0.982 |
| CD80 on granulocyte \|\| id:ebi-a-GCST90002040 | Inverse variance weighted | 3 | 0.202 | 0.154 | 0.189 | 1.224 | 0.905 | 1.656 |
| CD45 on CD33+ HLA DR+ CD14- \|\| id:ebi-a-GCST90002042 | Wald ratio | 1 | 0.061 | 0.220 | 0.782 | 1.063 | 0.691 | 1.636 |
| CD45 on CD33- HLA DR- \|\| id:ebi-a-GCST90002045 | Inverse variance weighted | 2 | 0.305 | 0.132 | 0.020 | 1.357 | 1.048 | 1.757 |
| CD45 on CD33- HLA DR+ \|\| id:ebi-a-GCST90002046 | Inverse variance weighted | 2 | -0.194 | 0.103 | 0.060 | 0.823 | 0.672 | 1.008 |
| CD45 on Immature Myeloid-Derived Suppressor Cells \|\| id:ebi-a-GCST90002052 | Inverse variance weighted | 2 | -0.134 | 0.082 | 0.103 | 0.875 | 0.745 | 1.027 |
| CD8 on Central Memory CD8+ T cell \|\| id:ebi-a-GCST90002054 | Inverse variance weighted | 3 | 0.162 | 0.126 | 0.198 | 1.176 | 0.919 | 1.506 |
| CD8 on naive CD8+ T cell \|\| id:ebi-a-GCST90002055 | Inverse variance weighted | 2 | 0.128 | 0.110 | 0.244 | 1.137 | 0.916 | 1.411 |
| CD8 on Effector Memory CD8+ T cell \|\| id:ebi-a-GCST90002056 | Inverse variance weighted | 4 | 0.103 | 0.095 | 0.280 | 1.108 | 0.920 | 1.335 |
| CD8 on Terminally Differentiated CD8+ T cell \|\| id:ebi-a-GCST90002057 | Inverse variance weighted | 2 | 0.095 | 0.117 | 0.415 | 1.100 | 0.875 | 1.384 |
| CD8 on CD8+ T cell \|\| id:ebi-a-GCST90002058 | Inverse variance weighted | 2 | 0.163 | 0.165 | 0.324 | 1.177 | 0.852 | 1.626 |
| CD8 on Natural Killer T \|\| id:ebi-a-GCST90002059 | Inverse variance weighted | 2 | 0.265 | 0.177 | 0.136 | 1.303 | 0.920 | 1.845 |
| CD8 on HLA DR+ CD8+ T cell \|\| id:ebi-a-GCST90002060 | Inverse variance weighted | 2 | 0.456 | 0.191 | 0.017 | 1.578 | 1.085 | 2.294 |
| CD4 on CD39+ CD4+ T cell \|\| id:ebi-a-GCST90002061 | Inverse variance weighted | 6 | 0.009 | 0.075 | 0.905 | 1.009 | 0.871 | 1.169 |
| CD4 on CD28+ CD4+ T cell \|\| id:ebi-a-GCST90002062 | Inverse variance weighted | 2 | 0.087 | 0.165 | 0.599 | 1.091 | 0.789 | 1.509 |
| CD4 on CD4 regulatory T cell \|\| id:ebi-a-GCST90002063 | Wald ratio | 1 | 0.089 | 0.192 | 0.644 | 1.093 | 0.750 | 1.594 |
| CD4 on resting CD4 regulatory T cell \|\| id:ebi-a-GCST90002064 | Wald ratio | 1 | 0.116 | 0.252 | 0.644 | 1.123 | 0.686 | 1.840 |
| CD4 on CD39+ resting CD4 regulatory T cell \|\| id:ebi-a-GCST90002065 | Wald ratio | 1 | -0.134 | 0.274 | 0.625 | 0.875 | 0.512 | 1.496 |
| CD4 on activated CD4 regulatory T cell \|\| id:ebi-a-GCST90002066 | Wald ratio | 1 | 0.087 | 0.188 | 0.644 | 1.091 | 0.755 | 1.575 |
| CD4 on CD39+ activated CD4 regulatory T cell \|\| id:ebi-a-GCST90002067 | Inverse variance weighted | 4 | 0.076 | 0.125 | 0.545 | 1.079 | 0.844 | 1.380 |
| CD4 on secreting CD4 regulatory T cell \|\| id:ebi-a-GCST90002068 | Wald ratio | 1 | 0.105 | 0.227 | 0.644 | 1.111 | 0.712 | 1.734 |
| CD4 on CD39+ secreting CD4 regulatory T cell \|\| id:ebi-a-GCST90002069 | Inverse variance weighted | 3 | -0.061 | 0.136 | 0.655 | 0.941 | 0.720 | 1.229 |
| CD4 on activated & secreting CD4 regulatory T cell \|\| id:ebi-a-GCST90002070 | Wald ratio | 1 | 0.091 | 0.196 | 0.644 | 1.095 | 0.745 | 1.609 |
| SSC-A on myeloid Dendritic Cell \|\| id:ebi-a-GCST90002071 | Inverse variance weighted | 7 | 0.049 | 0.171 | 0.773 | 1.051 | 0.751 | 1.469 |
| SSC-A on plasmacytoid Dendritic Cell \|\| id:ebi-a-GCST90002072 | Inverse variance weighted | 5 | 0.064 | 0.136 | 0.635 | 1.067 | 0.817 | 1.392 |
| SSC-A on monocyte \|\| id:ebi-a-GCST90002073 | Inverse variance weighted | 8 | 0.071 | 0.065 | 0.269 | 1.074 | 0.946 | 1.219 |
| SSC-A on CD14+ monocyte \|\| id:ebi-a-GCST90002074 | Inverse variance weighted | 6 | 0.065 | 0.071 | 0.363 | 1.067 | 0.928 | 1.228 |
| SSC-A on Natural Killer \|\| id:ebi-a-GCST90002076 | Wald ratio | 1 | 0.012 | 0.185 | 0.949 | 1.012 | 0.704 | 1.455 |
| SSC-A on HLA DR+ Natural Killer \|\| id:ebi-a-GCST90002077 | Inverse variance weighted | 3 | -0.232 | 0.101 | 0.022 | 0.793 | 0.651 | 0.967 |
| SSC-A on granulocyte \|\| id:ebi-a-GCST90002078 | Inverse variance weighted | 3 | -0.214 | 0.158 | 0.174 | 0.807 | 0.593 | 1.099 |
| SSC-A on lymphocyte \|\| id:ebi-a-GCST90002079 | Wald ratio | 1 | 0.312 | 0.296 | 0.292 | 1.366 | 0.765 | 2.439 |
| SSC-A on T cell \|\| id:ebi-a-GCST90002080 | Wald ratio | 1 | -0.462 | 0.443 | 0.298 | 0.630 | 0.264 | 1.503 |
| SSC-A on CD4+ T cell \|\| id:ebi-a-GCST90002081 | Inverse variance weighted | 4 | 0.158 | 0.145 | 0.274 | 1.172 | 0.882 | 1.556 |
| SSC-A on HLA DR+ T cell \|\| id:ebi-a-GCST90002083 | Wald ratio | 1 | -0.072 | 0.222 | 0.745 | 0.930 | 0.602 | 1.438 |
| SSC-A on Natural Killer T \|\| id:ebi-a-GCST90002084 | Inverse variance weighted | 2 | 0.065 | 0.218 | 0.764 | 1.068 | 0.697 | 1.636 |
| CD11c on myeloid Dendritic Cell \|\| id:ebi-a-GCST90002087 | Inverse variance weighted | 5 | 0.003 | 0.119 | 0.977 | 1.003 | 0.795 | 1.267 |
| CD11c on CD62L+ myeloid Dendritic Cell \|\| id:ebi-a-GCST90002088 | Inverse variance weighted | 2 | -0.013 | 0.105 | 0.904 | 0.987 | 0.803 | 1.214 |
| CD11c on monocyte \|\| id:ebi-a-GCST90002089 | Inverse variance weighted | 2 | -0.043 | 0.130 | 0.739 | 0.958 | 0.742 | 1.236 |
| CD11c on granulocyte \|\| id:ebi-a-GCST90002090 | Inverse variance weighted | 2 | -0.124 | 0.106 | 0.241 | 0.883 | 0.718 | 1.087 |
| CD11b on CD14+ monocyte \|\| id:ebi-a-GCST90002091 | Wald ratio | 1 | -0.027 | 0.067 | 0.690 | 0.974 | 0.854 | 1.111 |
| CD11b on Monocytic Myeloid-Derived Suppressor Cells \|\| id:ebi-a-GCST90002094 | Wald ratio | 1 | -0.036 | 0.091 | 0.690 | 0.964 | 0.807 | 1.152 |
| CD11b on CD33dim HLA DR- \|\| id:ebi-a-GCST90002095 | Wald ratio | 1 | 0.198 | 0.120 | 0.098 | 1.219 | 0.964 | 1.541 |
| CD11b on basophil \|\| id:ebi-a-GCST90002096 | Wald ratio | 1 | 0.192 | 0.116 | 0.098 | 1.212 | 0.965 | 1.522 |
| CD11b on CD33+ HLA DR+ CD14dim \|\| id:ebi-a-GCST90002097 | Wald ratio | 1 | -0.034 | 0.085 | 0.690 | 0.967 | 0.819 | 1.142 |
| CD45RA on naive CD4+ T cell \|\| id:ebi-a-GCST90002098 | Inverse variance weighted | 10 | -0.026 | 0.046 | 0.571 | 0.974 | 0.890 | 1.066 |
| CD4RA on Terminally Differentiated CD4+ T cell \|\| id:ebi-a-GCST90002099 | Inverse variance weighted | 3 | -0.028 | 0.060 | 0.636 | 0.972 | 0.864 | 1.093 |
| CD45RA on naive CD8+ T cell \|\| id:ebi-a-GCST90002100 | Inverse variance weighted | 2 | -0.346 | 0.157 | 0.027 | 0.707 | 0.520 | 0.962 |
| CD45RA on Terminally Differentiated CD8+ T cell \|\| id:ebi-a-GCST90002101 | Wald ratio | 1 | 0.524 | 0.258 | 0.042 | 1.688 | 1.019 | 2.797 |
| CD45RA on resting CD4 regulatory T cell \|\| id:ebi-a-GCST90002102 | Inverse variance weighted | 7 | -0.051 | 0.041 | 0.216 | 0.950 | 0.877 | 1.030 |
| CD45RA on CD39+ resting CD4 regulatory T cell \|\| id:ebi-a-GCST90002103 | Wald ratio | 1 | -0.131 | 0.089 | 0.141 | 0.877 | 0.736 | 1.044 |
| HLA DR on myeloid Dendritic Cell \|\| id:ebi-a-GCST90002104 | Inverse variance weighted | 7 | -0.090 | 0.051 | 0.075 | 0.914 | 0.828 | 1.009 |
| HLA DR on plasmacytoid Dendritic Cell \|\| id:ebi-a-GCST90002105 | Inverse variance weighted | 8 | -0.033 | 0.040 | 0.411 | 0.968 | 0.894 | 1.047 |
| HLA DR on Dendritic Cell \|\| id:ebi-a-GCST90002106 | Inverse variance weighted | 7 | -0.075 | 0.045 | 0.096 | 0.928 | 0.849 | 1.013 |
| HLA DR on CD33+ HLA DR+ CD14- \|\| id:ebi-a-GCST90002108 | Inverse variance weighted | 3 | -0.180 | 0.096 | 0.062 | 0.835 | 0.691 | 1.009 |
| HLA DR on CD33+ HLA DR+ CD14dim \|\| id:ebi-a-GCST90002109 | Inverse variance weighted | 3 | -0.250 | 0.134 | 0.062 | 0.779 | 0.600 | 1.012 |
| HLA DR on CD33dim HLA DR+ CD11b+ \|\| id:ebi-a-GCST90002110 | Inverse variance weighted | 4 | -0.049 | 0.067 | 0.470 | 0.952 | 0.835 | 1.087 |
| HLA DR on CD33dim HLA DR+ CD11b- \|\| id:ebi-a-GCST90002111 | Inverse variance weighted | 5 | -0.081 | 0.064 | 0.201 | 0.922 | 0.814 | 1.044 |
| HLA DR on CD33- HLA DR+ \|\| id:ebi-a-GCST90002112 | Inverse variance weighted | 3 | -0.066 | 0.051 | 0.194 | 0.936 | 0.847 | 1.034 |
| HLA DR on HLA DR+ T cell \|\| id:ebi-a-GCST90002113 | Wald ratio | 1 | 0.097 | 0.184 | 0.599 | 1.101 | 0.768 | 1.579 |
| HLA DR on HLA DR+ CD4+ T cell \|\| id:ebi-a-GCST90002114 | Wald ratio | 1 | 0.140 | 0.267 | 0.599 | 1.151 | 0.682 | 1.942 |
| HLA DR on B cell \|\| id:ebi-a-GCST90002116 | Inverse variance weighted | 9 | 0.030 | 0.062 | 0.633 | 1.030 | 0.912 | 1.164 |
| HLA DR on HLA DR+ Natural Killer \|\| id:ebi-a-GCST90002117 | Inverse variance weighted | 6 | -0.068 | 0.082 | 0.408 | 0.935 | 0.796 | 1.097 |
| CD8 on CD28+ CD45RA- CD8+ T cell \|\| id:ebi-a-GCST90002118 | Inverse variance weighted | 3 | 0.209 | 0.120 | 0.083 | 1.232 | 0.973 | 1.560 |
| CD8 on CD28+ CD45RA+ CD8+ T cell \|\| id:ebi-a-GCST90002119 | Inverse variance weighted | 8 | 0.072 | 0.074 | 0.332 | 1.074 | 0.930 | 1.241 |
| CD8 on CD28- CD8+ T cell \|\| id:ebi-a-GCST90002120 | Inverse variance weighted | 3 | -0.015 | 0.181 | 0.933 | 0.985 | 0.690 | 1.406 |
| CD8 on CD39+ CD8+ T cell \|\| id:ebi-a-GCST90002121 | Inverse variance weighted | 5 | -0.107 | 0.119 | 0.371 | 0.899 | 0.712 | 1.135 |
