# Supplementary material for: Exploring the Relationship Between Immune Cells and Scoliosis by Mendelian Randomization, Colocalization Analysis, and SMR
Source: Mediators Inflamm. 2025 Mar 26;2025:8833556. doi: 10.1155/mi/8833556 (PMC11964722; doi:10.1155/mi/8833556)
Supplement: Supporting Information 3 — Table S3: The significant results of univariable MR between the 40 immune cells and scoliosis. [file 8833556.f3.docx]

| exposure | method | nsnp | b | se | pval | or | or_lci95 | or_uci95 |
| --- | --- | --- | --- | --- | --- | --- | --- | --- |
| CD45RA on naive CD8+ T cell \|\| id:ebi-a-GCST90002100 | Inverse variance weighted | 2 | -0.346 | 0.157 | 0.027 | 0.707 | 0.520 | 0.962 |
| CD45RA on Terminally Differentiated CD8+ T cell \|\| id:ebi-a-GCST90002101 | Wald ratio | 1 | 0.524 | 0.258 | 0.042 | 1.688 | 1.019 | 2.797 |
| SSC-A on HLA DR+ Natural Killer \|\| id:ebi-a-GCST90002077 | Inverse variance weighted | 3 | -0.232 | 0.101 | 0.022 | 0.793 | 0.651 | 0.967 |
| CD8 on HLA DR+ CD8+ T cell \|\| id:ebi-a-GCST90002060 | Inverse variance weighted | 2 | 0.456 | 0.191 | 0.017 | 1.578 | 1.085 | 2.294 |
| CD45 on CD33- HLA DR- \|\| id:ebi-a-GCST90002045 | Inverse variance weighted | 2 | 0.305 | 0.132 | 0.020 | 1.357 | 1.048 | 1.757 |
| CD80 on monocyte \|\| id:ebi-a-GCST90002039 | Inverse variance weighted | 6 | -0.153 | 0.068 | 0.026 | 0.859 | 0.751 | 0.982 |
| HLA DR on CD14+ CD16+ monocyte \|\| id:ebi-a-GCST90002007 | Inverse variance weighted | 5 | -0.151 | 0.068 | 0.025 | 0.860 | 0.753 | 0.982 |
| CCR2 on CD14- CD16- \|\| id:ebi-a-GCST90002003 | Inverse variance weighted | 3 | 0.391 | 0.176 | 0.027 | 1.479 | 1.047 | 2.089 |
| PDL-1 on monocyte \|\| id:ebi-a-GCST90002002 | Inverse variance weighted | 2 | -0.260 | 0.129 | 0.044 | 0.771 | 0.599 | 0.993 |
| PDL-1 on CD14- CD16+ monocyte \|\| id:ebi-a-GCST90001999 | Inverse variance weighted | 2 | -0.245 | 0.113 | 0.030 | 0.783 | 0.628 | 0.976 |
| CX3CR1 on CD14- CD16- \|\| id:ebi-a-GCST90001994 | Inverse variance weighted | 3 | 0.355 | 0.138 | 0.010 | 1.427 | 1.088 | 1.870 |
| CCR2 on CD14+ CD16+ monocyte \|\| id:ebi-a-GCST90001992 | Inverse variance weighted | 2 | 0.704 | 0.268 | 0.009 | 2.022 | 1.196 | 3.417 |
| CD25 on secreting CD4 regulatory T cell \|\| id:ebi-a-GCST90001941 | Inverse variance weighted | 2 | -0.383 | 0.143 | 0.007 | 0.682 | 0.516 | 0.903 |
| CD25 on CD39+ secreting CD4 regulatory T cell \|\| id:ebi-a-GCST90001942 | Inverse variance weighted | 2 | -0.399 | 0.149 | 0.008 | 0.671 | 0.501 | 0.900 |
| CD25 on activated & secreting CD4 regulatory T cell \|\| id:ebi-a-GCST90001943 | Wald ratio | 1 | -0.416 | 0.192 | 0.030 | 0.660 | 0.453 | 0.962 |
| CD127 on CD8+ T cell \|\| id:ebi-a-GCST90001927 | Wald ratio | 1 | 0.522 | 0.233 | 0.025 | 1.686 | 1.068 | 2.661 |
| CD45 on Natural Killer \|\| id:ebi-a-GCST90001911 | Inverse variance weighted | 2 | 0.384 | 0.187 | 0.040 | 1.468 | 1.018 | 2.119 |
| CD86 on myeloid Dendritic Cell \|\| id:ebi-a-GCST90001903 | Wald ratio | 1 | 0.284 | 0.129 | 0.028 | 1.328 | 1.032 | 1.710 |
| CD28 on CD39+ CD8+ T cell \|\| id:ebi-a-GCST90001897 | Inverse variance weighted | 2 | 0.126 | 0.053 | 0.018 | 1.134 | 1.022 | 1.258 |
| CD16-CD56 on HLA DR+ Natural Killer \|\| id:ebi-a-GCST90001885 | Inverse variance weighted | 2 | 0.655 | 0.254 | 0.010 | 1.925 | 1.171 | 3.165 |
| CD25++ CD8+ T cell Absolute Count \|\| id:ebi-a-GCST90001681 | Inverse variance weighted | 3 | -0.308 | 0.113 | 0.007 | 0.735 | 0.589 | 0.918 |
| CD25++ CD8+ T cell %CD8+ T cell \|\| id:ebi-a-GCST90001680 | Wald ratio | 1 | -0.329 | 0.130 | 0.011 | 0.720 | 0.558 | 0.928 |
| CD28- CD8dim T cell %T cell \|\| id:ebi-a-GCST90001661 | Inverse variance weighted | 3 | -0.416 | 0.171 | 0.015 | 0.660 | 0.472 | 0.923 |
| CD14+ CD16- monocyte %monocyte \|\| id:ebi-a-GCST90001586 | Inverse variance weighted | 2 | 0.337 | 0.155 | 0.030 | 1.401 | 1.034 | 1.899 |
| CD16+ monocyte %monocyte \|\| id:ebi-a-GCST90001587 | Inverse variance weighted | 2 | -0.343 | 0.157 | 0.029 | 0.710 | 0.521 | 0.966 |
| CD45RA+ CD8+ T cell %T cell \|\| id:ebi-a-GCST90001562 | Inverse variance weighted | 3 | -0.316 | 0.153 | 0.038 | 0.729 | 0.540 | 0.983 |
| Terminally Differentiated CD8+ T cell %CD8+ T cell \|\| id:ebi-a-GCST90001558 | Inverse variance weighted | 3 | -0.275 | 0.132 | 0.037 | 0.760 | 0.587 | 0.983 |
| Terminally Differentiated CD8+ T cell %T cell \|\| id:ebi-a-GCST90001559 | Inverse variance weighted | 2 | -0.403 | 0.161 | 0.013 | 0.669 | 0.487 | 0.917 |
| Effector Memory CD4+ T cell Absolute Count \|\| id:ebi-a-GCST90001542 | Wald ratio | 1 | 0.327 | 0.150 | 0.029 | 1.386 | 1.034 | 1.859 |
| Effector Memory CD4+ T cell %CD4+ T cell \|\| id:ebi-a-GCST90001543 | Inverse variance weighted | 2 | 0.397 | 0.157 | 0.011 | 1.487 | 1.094 | 2.022 |
| Naive CD4+ T cell %CD4+ T cell \|\| id:ebi-a-GCST90001541 | Inverse variance weighted | 4 | -0.285 | 0.125 | 0.022 | 0.752 | 0.589 | 0.960 |
| CD45RA- CD4+ T cell %T cell \|\| id:ebi-a-GCST90001536 | Inverse variance weighted | 2 | 0.326 | 0.137 | 0.018 | 1.386 | 1.059 | 1.814 |
| CD45RA- CD4+ T cell Absolute Count \|\| id:ebi-a-GCST90001534 | Inverse variance weighted | 2 | 0.311 | 0.127 | 0.015 | 1.365 | 1.063 | 1.752 |
| Basophil %CD33dim HLA DR- CD66b- \|\| id:ebi-a-GCST90001533 | Inverse variance weighted | 3 | 0.143 | 0.073 | 0.050 | 1.154 | 1.000 | 1.330 |
| Basophil Absolute Count \|\| id:ebi-a-GCST90001532 | Inverse variance weighted | 3 | 0.076 | 0.036 | 0.036 | 1.079 | 1.005 | 1.159 |
| CD25++ CD45RA+ CD4 not regulatory T cell Absolute Count \|\| id:ebi-a-GCST90001507 | Inverse variance weighted | 3 | -0.307 | 0.104 | 0.003 | 0.736 | 0.600 | 0.902 |
| Plasmacytoid Dendritic Cell %Dendritic Cell \|\| id:ebi-a-GCST90001474 | Inverse variance weighted | 2 | -0.258 | 0.104 | 0.013 | 0.772 | 0.630 | 0.946 |
| Myeloid Dendritic Cell %Dendritic Cell \|\| id:ebi-a-GCST90001459 | Inverse variance weighted | 2 | 0.224 | 0.103 | 0.030 | 1.251 | 1.022 | 1.531 |
| IgD- CD38dim B cell %lymphocyte \|\| id:ebi-a-GCST90001426 | Inverse variance weighted | 2 | -0.578 | 0.231 | 0.012 | 0.561 | 0.357 | 0.882 |
| IgD+ CD38+ B cell %lymphocyte \|\| id:ebi-a-GCST90001429 | Wald ratio | 1 | 2.438 | 1.238 | 0.049 | 11.449 | 1.011 | 129.651 |
